# Novel PEG_6000_–Silica-MWCNTs Shape-Stabilized Composite Phase-Change Materials (ssCPCMs) for Thermal-Energy Storage

**DOI:** 10.3390/polym15143022

**Published:** 2023-07-12

**Authors:** Cristina Lavinia Nistor, Ioana Catalina Gifu, Elena Maria Anghel, Raluca Ianchis, Cristiana-Diana Cirstea, Cristian Andi Nicolae, Augusta Raluca Gabor, Irina Atkinson, Cristian Petcu

**Affiliations:** 1 Polymers Department, National Institute for Research and Development in Chemistry and Petrochemistry-ICECHIM, 202 Spl. Independentei, 060021 Bucharest, Romania; cristina.nistor@icechim-pd.ro (C.L.N.); catalina.gifu@icechim-pd.ro (I.C.G.); raluca.ianchis@icechim-pd.ro (R.I.); cristian.nicolae@icechim.ro (C.A.N.); raluca.gabor@icechim.ro (A.R.G.); 2 Institute of Physical Chemistry “Ilie Murgulescu” of the Romanian Academy, 202 Splaiul Independentei, 060021 Bucharest, Romania; irinaatkinson@yahoo.com; 3 National Institute for Research and Development in Electrical Engineering ICPE-CA, INCDIE ICPE-CA, 313 Splaiul Unirii Street, 030138 Bucharest, Romania; diana.cirstea@icpe-ca.ro

**Keywords:** shape-stabilized phase change materials, PEG-silica PCMs, energy storage, latent heat, thermal properties, solid–liquid phase change

## Abstract

This paper describes the preparation of new PEG_6000_–silica-MWCNTs composites as shape-stabilized phase change materials (ssPCMs) for application in latent heat storage. An innovative method was employed to obtain the new organic–inorganic hybrid materials, in which both a part of the PEG chains, used as the phase change material, and a part of the hydroxyl functionalized multiwall carbon nanotubes (MWCNTs-OH), used as thermo-conductive fillers, were covalently connected by newly formed urethane bonds to the in-situ-generated silica matrix. The study’s main aim was to investigate the optimal amount of PEG_6000_ that can be added to the fixed sol–gel reaction mixture so that no leakage of PEG occurs after repeated heating–cooling cycles. The findings show that the optimum PEG_6000_/NCOTEOS molar ratio was 2/1 (~91.5% PEG_6000_), because both the connected and free PEG chains interacted strongly with the in-situ-generated silica matrix to form a shape-stabilized material while preserving high phase-transition enthalpies (~153 J/G). Morphological and structural findings obtained by SEM, X-ray and Raman techniques indicated a distribution of the silica component in the amorphous phase (~27% for the optimum composition) located among the crystalline lamellae built by the folded chains of the PEG component. This composite maintained good chemical stability after a 450-cycle thermal test and had a good storage efficiency (~84%).

## 1. Introduction

In response to several international regulations regarding cost-optimal minimum energy performance requirements, thermal energy storage has emerged as a promising method for energy-efficient use. Thus, energy storage plays a crucial role in saving excess energy and resolving the imbalance between energy supply and demand [1,2]. For renewable energies such as solar and geothermal, energy storage helps to store a huge amount of energy that otherwise can be lost [3,4,5,6]. In solar thermal applications, collectors are employed to capture thermal sun radiations for home and water heating [7,8]. One popular option for the recovery of solar energy has been the use of PCMs as part of heat exchangers [9]. In addition, energy storage can help reduce energy consumption and CO_2_ emissions in buildings and industries [10]. Due to their superior thermal storage capabilities, phase change materials (PCMs) have enormous promise for latent thermal energy storage, waste heat recovery, heating, and cooling systems [11]. Through melting and solidification, they either absorb or release energy. Additionally, PCMs for latent heat storage provide the benefits of sustainability and environmental preservation, enabling more intelligent use of energy [12]. Thus, PCMs are widely used in many applications, including the use of solar energy, energy-efficient construction, electronics heat management, waste-heat recovery, and more [13,14].

Over the past two decades, many studies were reported on latent heat storage with solid–liquid phase change materials [15,16]. Among the latter materials, organic PCMs, which are divided into paraffins and non-paraffins, can be prepared using both physical methods (adsorption, mixing, and solvent evaporation) and chemical methods (cross-linking and in situ co-polymerization) [17]. When chemically creating organic composites with PCMs, one polymer component acts as a solid matrix—usually a thermoset component—while a second polymer component (thermoplastic component) that acts as a heat storage medium is attached to the matrix via chemical bonds. While the polymer system is still in the solid state during the phase transition, the crystalline polymer stores or releases energy [18].

Polyethylene glycols (PEGs) are a particular category of solid–liquid non-paraffinic PCMs with high latent heat, low toxicity or corrosion, strong chemical stability, and low cost. The semicrystalline polyethylene glycols exhibit phase transitions in a variety of thermal ranges (−55 °C to 100 °C), depending on the length of their molecular chains. However, they have poor thermal conductivity and need to be placed in sealed containers due to the leakage that occurs during melting. Except for leakage prevention during solid–liquid phase transition of a PEG, its covalent binding into a matrix is required for certain applications, such as Li-ion batteries [19,20,21]. PEGs’ extremely low thermal conductivity is one of the greatest drawbacks that must be addressed. Fins, metallic fiber, and metal foams [22] have been used to enhance this property, in addition to the incorporation of nano- and micro-additives such as metal-oxides [23], expanded graphite [24], graphene [25], graphite foam, carbon foam matrix, and carbon nanotubes (CNTs) [7,12,26,27,28]. Moreover, reduced load capacity and significantly lower latent heat values might hinder the use of these materials. Multifunctional composites have been obtained when CNTs were selected as fillers for phase-change composites. Thus, CNT-containing PCMs bring the advantage of energy conversion (light-to-heat conversion) along with thermal energy storage [29]. CNTs as hosts for PCMs should meet some requirements, including open ends, a larger diameter/length ratio, and very good wettability [30]. Moreover, higher thermal conductivity in the longitudinal direction is expected in the case of composites containing single-wall carbon nanotubes (SWCNTs), in contrast to the multi-wall carbon nanotubes, (MWCNTs) [30]. This means that arrayed CNTs show higher thermal conductivity than random CNTs composites. In addition, the functionalization of the CNTs hinders agglomeration in the host matrix. Covalent binding of NH_2_-functionalized MWCNT filler polymers has been considered [31]. Hence, the inhomogeneous dispersion and random orientation of various CNT fillers in PCM composites and the prevention of their sedimentation during repeated heating–cooling cycles are challenging matters to solve.

Although shape-stabilized PCM composites (ssPCMs) have been reported previously, they usually refer to phase-transition materials that are impregnated in porous substrates or encapsulated in polymer or hybrid microparticles [32] and/or to branched polymers in which the main chains are polymerized between themselves, providing the stabilized structure with implications for the crystallinity of the thermoplastic component and, hence, for phase-transition characteristics.

In a survey of the literature, we discovered numerous research articles on the development of PEG-silica ssPCMs, most of which are obtained by impregnating the PCM in a silica porous support [24,33,34,35,36,37]. As far as we know, only a small number of reported studies deal with development of an ssPCM prepared by an in situ sol–gel chemical method [38,39,40,41,42,43,44]. Thus, He et al. [38] showed that PEG was dispersed into the in-situ-generated mesoporous silica and formed stable core–shell structures. Serrano et al. [39] reported that in the sol–gel-derived ssPCMs, PEG either functioned in a stable PEG-SiO_2_ matrix without latent heat or was adsorbed onto the matrix while preserving latent-heat performances. Young et al. [40] prepared PEG-based ssPCMs by the sol–gel method through the hydrolysis of tetraethylorthosilicate (TEOS) in acidic medium. They showed that the phase competition and crystallization behavior of PEGs under various confinements of SiO_2_ can be used to explain the various thermodynamic behaviors of the various composites they investigated.

In this work, we present an innovative approach to synthesize new ssCPCMs, in which both a part of the PEG chains, used as the phase changing material, and a part of the hydroxyl functionalized multiwall carbon nanotubes (MWCNTs-OH) fillers were covalently connected by newly formed urethane bonds to the in-situ-generated silica matrix. Samples with various molar ratios of PEG_6000_ and 3-(triethoxysilyl)propyl isocyanate (NCOTEOS) coupling agent (1/2, 1/1, 2/1, and 3/1) were used for the sol–gel preparation of novel ssCPCMs. The main objective of this study was to determine the optimum amount of PEG_6000_ that may be added to the sol–gel reaction mixture to prevent PEG leakage from the composite during multiple heating–cooling cycles. The effect of covalent coupling of the MWCNTs-OH fillers on morphology, thermal, and mechanical characteristics of the obtained composites was also investigated.

## 2. Materials and Methods

### 2.1. Materials

Polyethylene glycol (synthesis grade) with a molecular mass of ~6000 g/mol (PEG_6000_) was purchased from Scharlau (Scharlab S.L., Sentmenat, Barcelona, Spain). The surfactant, sodium dodecyl sulfate ≥98.0% (SDS), was provided by Fluka (Sigma–Aldrich Chemie GmbH, Buchs, Switzerland). The 1,4-diazabicyclo[2.2.2]octane (DABCO)–synthesis grade (Merck–Schuchardt, Hohenbrunn bei München, Germany) has the role of catalyzing the coupling reaction of PEG_6000_ with the silica co-precursor 3-(Triethoxysilyl)propyl isocyanate 95.0% (NCOTEOS)–Sigma–Aldrich (Sigma–Aldrich Co, St. Louis, MO, USA). Multiwall carbon nanotubes functionalized with hydroxyl groups (MWCNTs-OH) with an inner diameter of 50 ± 20 nm and a length of 1–15 μm (FutureCarbon GmbH, Bayreuth, Germany) were added as thermoconductive fillers. The used silica co-precursors were tetraethylorthosilicate (TEOS) ≥99.0% (Sigma–Aldrich Chemie GmbH, Steinheim, Germany); (3-Glycidyloxypropyl)trimethoxysilane (GPTMS) ≥98.0%–Aldrich (Sigma–Aldrich Chemie GmbH, Steinheim, Germany) and methyltrimethoxysilane (MeTMS) 97.0% (Acros Organics, NJ, USA). Ammonia solution 25% (NH_4_OH 25%) (CHIMREACTIV S.R.L, Bucharest, Romania) was used to catalyze the sol–gel reactions between the silica co-precursors and to provide the water needed in the hydrolysis reaction. n-Octane purum (LOBA Feinchemie GmbH, Fischamend, Austria) was the solvent used for the emulsification of the PEGs. Absolute ethyl alcohol (EtOH) ≥99.5% (CHIMREACTIV S.R.L, Bucharest, Romania) was the solvent used for the silica co-precursors; it also acted as the emulsifying agent for the PEGs in n-octane.

### 2.2. Preparation of PEG_6000_–Silica-MWCNTs ssCPCMs

The new ssCPCMs (labeled TP0, TP2, TP3, TP4, TP5, and TP15) were prepared by the sol–gel synthesis route, at four different PEG_6000_/NCOTEOS molar ratios (1/2, 1/1, 2/1, and 3/1) corresponding to various concentrations of PEG_6000_ in the dried final composite (Table 1). While the amount of PEG fluctuated, the amounts of silica and the filler (MWCNTs-OH) were kept constant (0.02%) for all the samples, except for the TP15 sample, where a larger amount (0.32%) of filler was used for comparison reasons.

A mechanical stirrer (STIRRER DLS, VELP Scientifica Srl, Usmate, Italy) was used to homogenize samples TP2–TP4 that were prepared for this study. The water bath was heated on an AREX 6 Heating Magnetic Stirrer (VELP Scientifica Srl, Usmate, Italy) equipped with a VTF Digital Thermoregulator (VELP Scientifica Srl, Usmate, Italy). The resulting material, after drying, was ground by hand, then pressed using a 15 ton manual press (Specac Ltd., Orpington, UK) in a Specac mold (13 mm Evacuable Die and a 13 mm Disk Holder), which is usually used to obtain KBr pellets for FT–IR.

**Scheme 1:**
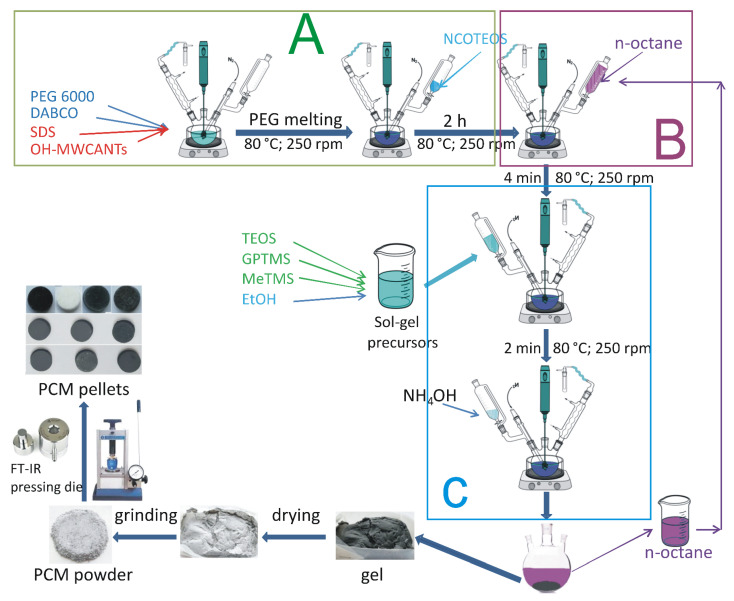
Bubbling and feeding funnels were introduced: (i) the required amount of PEG_6000_ (Table 2); (ii) the required amount of MWCNTs-OH; (iii) 4 g of surfactant (SDS) required for an improved dispersion of CNT in; and (iv) 1,4-diazabicyclo[2.2.2]octane (DABCO) in a proportion of 1% by weight relative to the amount of PEG_6000_. DABCO has a catalytic role in the coupling reaction between PEG_6000_ and MWCNTs-OH with NCOTEOS.

The synthesis of these new shape-stabilized phase-change materials, with thermal-energy storage properties, involved three reaction mixtures (A, B, and C, Table 2), whose addition corresponded to the following stages (A, B and C; Scheme 1):

*Stage A. Covalent connection of PEG and MWCNTs with the silica co-precursor NCOTEOS (preparation of PEG_6000_-Si and MWCNT-Si)*. During this stage, a portion of the PEG_6000_ chains formed covalent bonds with the silica co-precursor 3-(triethoxysilyl)propyl isocyanate (NCOTEOS). As a result, some PEG chains were able to create covalent connections with the silica network that subsequently formed in the presence of the silica co-precursors mixture (Stage C). This connection was aimed at stopping the PEG from leaking out of the final composite structure during numerous heating–cooling cycles.

At the same time as the formation of the PEG_6000_–Si silica building block, the connection of the OH-functionalized multi-walled carbon nanotubes (MWCNTs-OH) with NCOTEOS occurred in this step, with the goal of preventing their deposition on the bottom of the flask and favoring their homogeneous distribution in the finished PCM composite by covalently bonding the MWCNTs with the in-situ-formed silica network.

Following the reaction between the hydroxyl groups of the two components with the isocyanate functions of NCOTEOS, we obtained PEG_6000_ and MWCNTs end-capped with Si(O-CH_2_-CH_3_)_3_ sol–gel reactive functions. The new building blocks, denoted PEG_6000_-Si and MWCNTs-Si, respectively, were to be used for the construction of the three-dimensional (3D) macromolecular network structure that would be produced in situ.

The flask containing all four of these ingredients was put into a water bath that was heated to 80 °C. NCOTEOS was added to the flask only after the PEG had completely melted and SDS had fully dissolved. Both PEG_6000_ and MWCNTs-OH reacted with NCOTEOS in a coupling reaction that lasted for two hours at 80 °C under 250 rpm mechanical stirring in an inert nitrogen environment. Tao et al. previously reported the useful application of SDS as a surface active agent for a better dispersion of CNTs in PEG [45]. At this moment, we anticipated that PEG_6000_-Si silica building blocks could be found in the flask, arising from the coupling of PEG_6000_ with NCOTEOS, free PEG_6000_, thermoconductive filler coupled with NCOTEOS (MWCNT-Si), and possibly free thermoconductive filler. Combining these components resulted in mixture A.

*Stage B. Obtaining inverse emulsion of PEG in n-octane*. The mixture A from the flask was combined with 80 mL of n-octane (mixture B) that was preheated to 70 °C. The newly formed mixture was mechanically agitated at 250 rpm while being kept at a temperature of 80 °C under an inert nitrogen environment. Shortly after n-octane was poured over mixture A, phase separation could be observed, revealing a thick black layer and a thin clear layer at the top.

*Stage C.* In situ *synthesis by sol–gel process of the SiO_2_ network.* Using a feeding funnel, a mixture of 5 g TEOS, 0.567 g GPTMS, 0.327 g MeTMS, and 20 mL EtOH (molar ratio TEOS/GPTMS/MeTMS = 10/1/1) was slowly added to the flask. When the combination of sol–gel precursors dissolved in ethanol was added, the two phases existing in the flask began to homogenize. The presence of ethanol assisted in emulsifying the PEG in n-octane. 1.5 mL of NH_4_OH (25%) was added after the mixture in the flask had completely homogenized (for about two minutes) and the pH was adjusted to 7.

The reaction continued under mechanical stirring at 250 rpm, at 80 °C and under nitrogen atmosphere until coagulation appeared or, in the case of sample TP5, for 3 h. Approximately 1 h after the NH_4_OH addition, two phases could be seen to be separated in samples TP2, TP3, and TP4, and the stirring was ended. A dark solid phase was formed at the bottom of the flask that had a gel-like appearance and a liquid clear phase at the top. After the clear phase was eliminated, the resulting gel was put into a polyethylene tray and allowed to air dry for 48 h at room temperature. The TP5 sample remained homogeneous even 3 h after the NH_4_OH addition, without the phase separation observed in the other samples. Because there was an excessive amount of PEG in this sample, the silica network was unable to form properly, which prevented PEG from coagulating. As all samples were prepared at 80 °C, the PEG from the flask existed in the melted state, so all the composites during preparation were soft and could flow, even after coagulation. Immediately after observing the occurrence of the coagulation, and while the obtained materials were still warm and soft, the samples were poured from the flask into polyethylene trays, where, as they cooled, they hardened due to the solidification of the PEG. They were then allowed to dry at room temperature for at least 48 h before different specimens were removed for various characterizations.

### 2.3. Methods

SEM images were recorded with an ESEM-FEI Quanta 200 Scanning electron microscope (FEI Company, Eindhoven, The Netherlands). The working parameters used in the study of the materials through SEM were high vacuum, an LFD detector, and magnification between 50×–5000×. For their morpho-structural characterization, the materials were also examined by scanning electron microscopy (SEM) and energy-dispersive X-ray spectroscopy (EDX), which analyzes the composition of the elements’ distinctive X-rays. A field-emission scanning electron microscope, Hitachi TM4000plus II (Hitachi High-Technologies Corp Ltd., Tokyo, Japan), which was connected to an energy-dispersive X-ray spectrometer, was used. EDX was used to evaluate the distribution of C, O, and Si elements along a scan line in the analyzed samples. A BSE detector was employed at an accelerating voltage of 20 kV and a magnitude of 500 X.

Preparing the samples for this study involved taking a small piece of each sample and depositing it on a carbon strip and covering it with a 5 nm layer of Au (through sputter coating). For each sample, both SEM pictures and EDAX spectra were acquired. X-ray diffractograms were recorded within the range of 5° to 70° (2θ) with a Rigaku diffractometer, type Ultima IV (Rigaku Corporation, Tokyo, Japan), equipped with a Cu tube operating at 40 kV and 30 mA. The crystallinity degree of the PEG 6000 component was obtained by fitting the defined X-ray diffraction peaks over the range of 16 to 30° (2θ), as described elsewhere [46].

UV-Raman spectra were collected by means of a LabRam HR800 spectrometer (Horiba France SAS, Palaiseau, France) equipped with a 325 nm laser line (Kimmon Koha Co., Ltd., Tokyo, Japan), a grating of 2400 lines, and a 40×/0.47 NUV objective (Olympus Corporation, Tokyo, Japan). The Edge filter prevented recording Raman signal at low wavenumbers. Silicon was used for peak position calibration. The laser power (P_L_) was adjusted to avoid sample heating and degradation (P_L_ = 5, 1.5 and 0.5 mW).

FTIR spectra were obtained with a Bruker Tensor 37 spectrometer (Bruker Instruments, Woodstock, NY, USA). The analysis parameters were 32 scans with a resolution of 4 cm^−1^ in the wavelength range of 4000 to 600 cm^−1^. Samples were analyzed using the attenuated total reflection (ATR) module with a Golden Gate diamond unit (400–4000 cm^−1^).

The thermal stability of the composite material was studied in the temperature range of 0 to 700 °C. TGA analysis was performed using a Q5000 apparatus from TA Instruments (New Castle, DE, USA). The mass of the samples varied between 10 and 15 mg. These were placed in 100 µL platinum pans. All TGA experiments were conducted in a nitrogen environment with a purge flow rate of 40.0 mL/min. The TGA program consisted of heating up to 710 °C, with a 10 °C/min increment, followed by a 5 min. isotherm.

DSC measurements were performed using a DSC Q2000 apparatus from TA Instruments (New Castle, DE, USA). DSC runs were carried out on powders of about 10 mg encapsulated in Tzero^®^ aluminum pans. The purge gas used was helium, at a flow rate of 5 mL/min. The DSC method used to analyze the thermal properties of the selected samples was a dynamic method, consisting of a cooling process with a rate of 10 °C/minute up to −10 °C, an isotherm of 3 min followed by a heating with 10 °C/min up to 80 °C, and another 3 min isotherm for sample stabilization. This run was repeated three times (3 heating–cooling cycles) for all samples and 450 times for sample TP4. Indium was used as the reference for calibration of melting temperature (extrapolated onset melting temperature of 156.49 ± 0.01 °C, obtained with onset values recorded at various heating rates of 2, 5, 10, and 20 °C) and enthalpy (28.27 ± 0.07 J/g).

The analysis of the thermo-physical properties (thermal diffusivity, specific heat capacity, effusivity, and thermal conductivity) were carried out by the “flash method”, according to ASTM E 1461 [47], using an Netzsch LFA 447 Nanoflash device (Netzsch, Selb, Germany). The LFA equipment allowed the direct measurement of the thermal diffusivity, while the specific heat of the materials was determined by a differential method, using a reference sample. Two mathematical models, the Adiabatic and Cowan models, both with linear baseline correction, were used for the analysis of the data. The analysis included the possibility of heat loss from both the front and back faces of the sample. It was also assumed that the energy pulse was rectangular in shape and of relatively short duration. For the specific heat measurement, the magnitude of the temperature rise of an unknown sample was compared to that of the pyroceram standard. The device could perform tests in the temperature range of 25 to 300 °C, for cylindrical samples with a diameter of 12.7 mm and a thickness of 2–3 mm. A high-performance xenon lamp was used as a source of radiation energy, and the irradiation time on the front face of the sample was 0.18 ms. A minimum of five measurements were performed for each sample at a temperature of 25 °C. The increase in temperature on the other surface of the sample was measured using an infrared (IR) InSb detector, cooled with liquid nitrogen.

Thermo-mechanical properties were analyzed with a DMA Q800 V20 instrument (TA Instruments, New Castle, DE, USA). The working conditions were a DMA controlled force mode, the stress-strain method, air-bearing gas, and air.

## 3. Results and Discussions

### 3.1. Leakage Test

**Figure 1:**
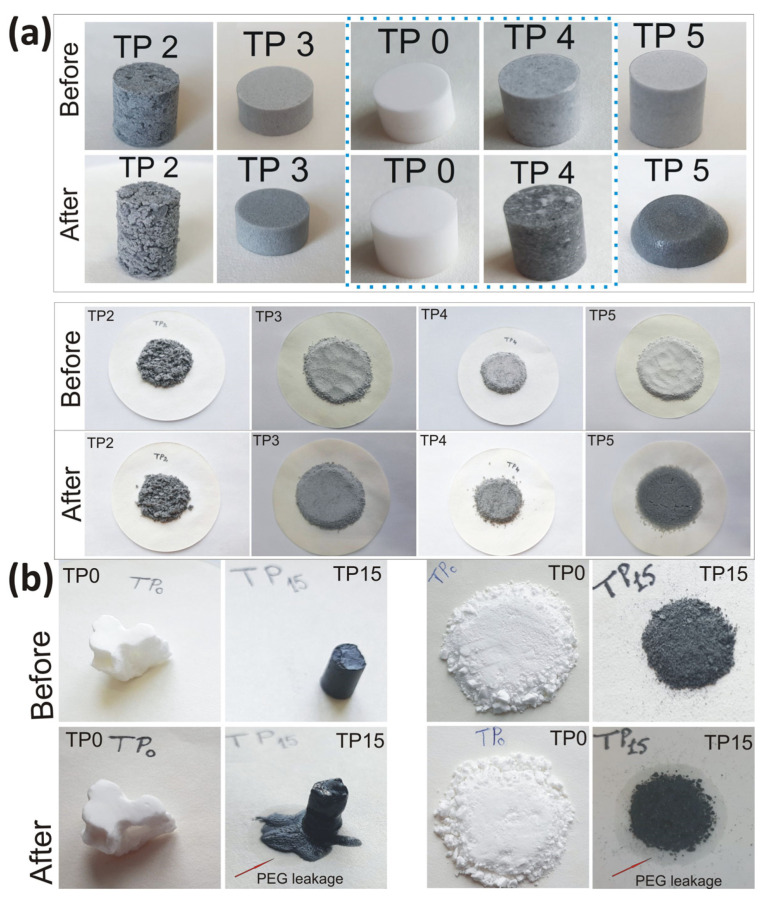
Leakage test of the (**a**) TP(2/3/4/5) and (**b**) PT(0/15) composites. The appearance of the samples is shown before and after 2 h of exposure to 85 °C. The irregular-shaped TP0 and TP15 specimens were obtained by pouring samples immediately at the end of the synthesis process.

After 48 h of drying, both ground and cylinder-shaped samples were placed on circular filter papers with a diameter of 10 cm. Images of the samples taken prior to the thermal test are displayed in the upper part of Figure 1. In order to determine whether PEG_6000_ flowed from the hybrid material in which it was integrated and whether it moistened the filter paper, the samples were put in an oven and heated for two hours at a temperature of 85 °C (far above the melting point of PEG_6000_, which is 61.4 °C, as shown in Table 3).

As can be seen from Figure 1a (images collected after leakage test), PEG leakage occurred for none of the ground samples except for the TP5 and TP15 composites. In the case of the TP5 sample (which contained the highest percentage of PEG_6000_), a slight wetting of the filter paper was visible. This was worsened in the case of PT15, with the highest content of MWCNTs-OH (Figure 1b). Instead, the carbon nanotubes-free sample, PT0, easily passed the leakage test.

In addition to the leakage test conducted on ground samples, we also performed leakage tests on cylindrical specimens. These specimens were heated in an oven for 2 h at 80 °C. The time allocated to this test was sufficient to allow us to observe the leakage and shape deformation of the two samples with low shape-stabilized performances (TP5 and TP15). For the other samples (TP2, TP3, TP4, and TP0), no leakage or shape deformation occurred. In addition, for samples TP5 and TP15, a strong tendency to impregnate the filter paper on which they were placed during testing and to stick to it after cooling was observed. In contrast, samples TP2, TP3, TP4, and TP0 did not wet the paper and could be easily detached. However, the cylinder made from the TP2 composite was observed to be very brittle, especially after heating, thus limiting the usability of this composite. This friability was also confirmed by DMA analysis, as discussed below. Both the TP4 and TP3 samples exhibited very good mechanical and shape-stabilized performances, but after the evaluation of the thermophysical properties, we selected TP4 to check its long–term reliability (via a cycling test), because it exhibited the highest transition enthalpy (i.e., the highest latent-heat storage capacity).

### 3.2. Evaluation of the Thermal Energy Storage Capacity for the Obtained Materials

Differential scanning calorimetry (DSC) was used to assess thermal performances, e.g., the enthalpy and temperature of phase transitions, of pristine and PEG_6000_-based materials that had heat-storage properties.

**Figure 2:**
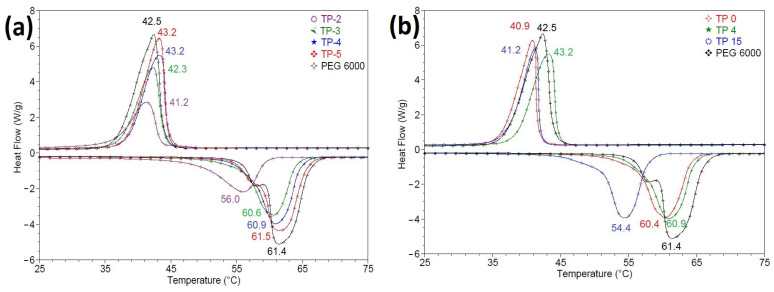
DSC diagrams corresponding to the third heating–cooling cycle of (**a**) TP(2/3/4/5) composites, obtained with various PEG_6000_/NCOTEOS ratios, and (**b**) TP(0/4/15) composites, obtained with the same PEG_6000_/NCOTEOS ratio, but with various amount of MWCNTs.

The DSC curves, the melting and crystallization temperatures, and the melting and crystallization enthalpies for the PEG_6000_ and TP(0/2/3/4/5/15) samples are displayed in Figure 2 and Table 3, respectively. PEG_6000_ was selected as the phase-change material for this work because of its high value of melting and solidification enthalpies (Table 3) and its phase-transition temperatures of T_m_ = 61.4 °C and T_s_ = 42.7 °C (Figure 2a), which were suitable for various applications in thermal-energy storage.

Single asymmetric melting and solidification effects were obtained for composite materials, in contrast with the pristine PEG_6000_ with multiple-melting effects. Decreases in the melting temperature of the PEG_6000_ component in the TP(0/2/3/4/15) composites might have been due to three factors: (i) covalently and weak interaction with SiO_2_, (ii) PEG confinement, and (iii) thermal-conductivity modification [49,50]. Even weak PEG-SiO_2_ interaction through capillary forces, hydrogen bonding, and surface forces causes a lowering of melting points [49]. The subcooling (ΔT values in Table 3) of the PEG component (calculated as the difference between the melting and solidification temperatures), which is detrimental for long-lasting heat storage purposes, was lowered for the composite materials obtained in this study, except for the MWCNT-free sample, TP0. The competition of these two processes (the “confining effect” [51] and covalent bonding), which restricted the growth to the stable phase (crystal) of the PEG, can explain the different behavior of the samples when the PEG content increased.

According to the high enthalpies of melting and solidification, exceeding 90 J/g, a reasonable amount of heat could be stored and released by these materials. However, these values were lower than the theoretical ones. Additionally, the PEG_6000_ degree of coupling to the inorganic network, which is required to stabilize the shape of the material during the subsequent melting–solidification cycles, was partly to blame for the decrease in the enthalpy values recorded for the composite materials, in comparison to the pristine PEG. A relative enthalpy-efficiency coefficient was introduced to describe thermal energy storage capacity, according to the following equation [52]:

(1) $$\lambda_{m\;}(\%)=\frac{{\Delta H}_{m}}{{\Delta H}_{mPEG6000\;}\times w_{PEG6000}}\times 100$$

where

*λ_m_* = phase-transition capacity for the heating stage (the relative enthalpy efficiency);

Δ*H_m_* = melting enthalpy of ssPCM;

Δ*H_mPEG_*_6000_ = melting enthalpy of pure PEG_6000_;

*w_PEG_*_6000_ = percentage of PEG_6000_ in the final material.

The enthalpy-efficiency coefficient was greatly diminished (65.83% and 66.77%) for the TP2 sample, with the lowest PEG_6000_ content. This difference was determined by the different interaction of PEG within the silica framework [40,52] and the crystallization behavior of the PEG chains, i.e., the crystallization degree and arrangement of the chains [46,49,50,51,52,53,54]. The enthalpy-calculated crystallization degree, χ_c_ (see Table 3) showed the smallest values for the TP2 (67.17%) and TP15 (78.87%) composites. In addition, the crystallization degree was calculated by integration of the X-ray patterns as presented in the following results.

### 3.3. Structure and Morphology of the Semicrystalline PEG_6000_-Based Composites

**Figure 3:**
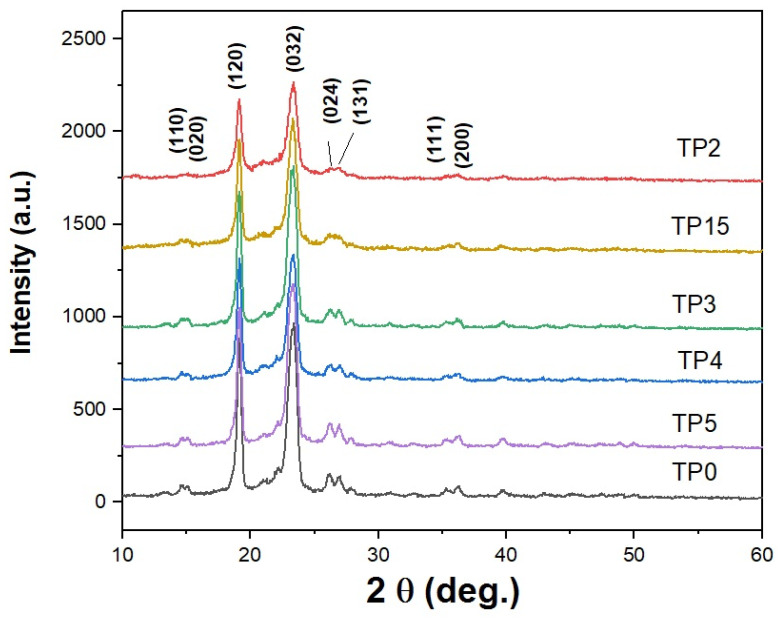
X-ray diffraction patterns of the TP(0/2/3/4/5/15) composites.

The n-folded (n stands for 0, 1, and 2) chains of semicrystalline PEG_6000_ form lamellae and, further, build spherulites [49,51]. The diffractograms of the TP(0/2/3/4/5/15) composites shown in Figure 3 are dominated by sharp peaks that are assignable to the crystalline phase of the PEG_6000_ component, with the two main peaks at about 19.13 and 23.36° and with an amorphous halo, due to amorphous counterpart of PEG and silica.

The calculated d-inter-reticular spacing values of the TP0, TP4, and TP15 samples prepared with the same PEG_6000_/NCOTEOS ratio and variable amounts of MWCNTs-OH were comparable to those reported in the previous study for the PEG_6000_/epoxy composite [46] of 0.46 (d_120_), 0.38 (d_032_), 0.34 (d_024_), and 0.33 nm (d_131_) corresponding to X-ray peaks at ~19, 23, 26, and 27°, respectively.

Analogous to the PEG_6000_/epoxy composite (70/30), the more intense peak of PEG_6000_ at ~27°, corresponding to a larger crystallite size (24.41 nm) in a direction perpendicular to the (131) plane [46], was diminished for the TP(0/4/15) composites, very likely due to restrained thickening of the PEG lamellae under silica and/or filler action.

Wider and less-intense peaks were recorded for TP2 and TP15 and, hence, their crystallinity degree (see Table 3) was the lowest among the composites presented here. Good agreement between the DSC and XRD-derived crystallinity was obtained for all composites in this work.

**Figure 4:**
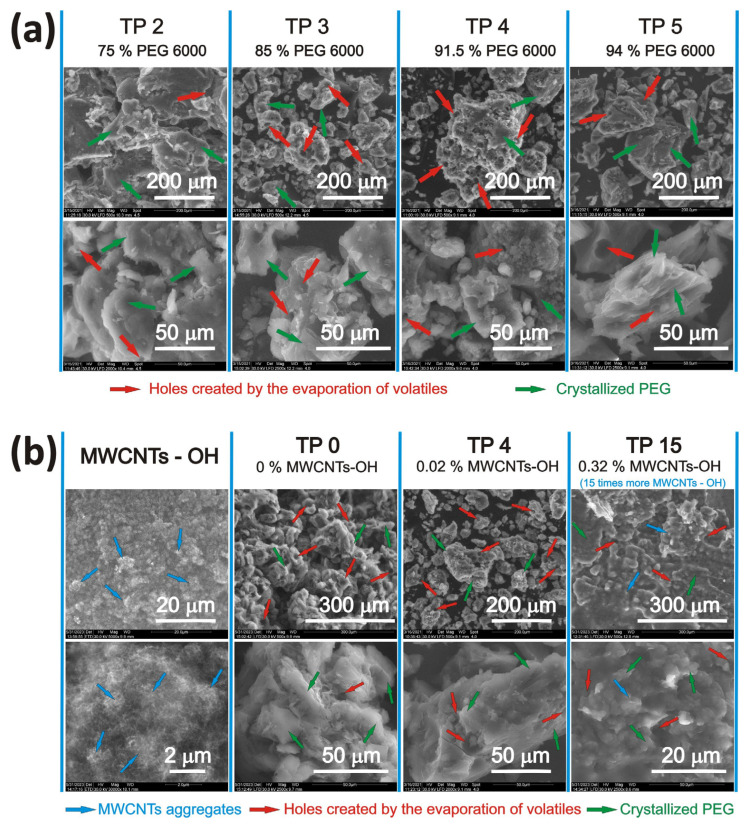
SEM images of the samples with increasing content of (**a**) PEG_6000_ component and (**b**) MWCNTs-OH filler (the same PEG_6000_/NCOTEOS ratio of 2/1 was used for preparation of TP(0/4/15)). Aggregates of the MWCNTs-OH filler are used for comparison.

The SEM micrographs in Figure 4 show different morphology, in accordance with the PEG_6000_/NCOTEOS ratio (see Table 1) and the MWCNTs-OH amount used in preparation of the PT(0/2/3/4/5/15) composites. Lamellae morphology is easier to depict in the Figure 4a and Figure S1 from the TP2 to TP5. TP5 showed richer folds for pristine PEG_6000_ that form spherulites [53]. Slightly different chain folding and/or lamellae wrapping was noticeable for the TP3 and TP4 samples.

The MWCNTs-OH are densely entangled in Figure 4b. Increasing MWCNTs-OH content at the same PEG_6000_/NCOTEOS ratio used in preparation of the PT(0/4/15) samples triggered the formation of globular structures in the case of PT15 (Figure 4b).

According to the EDX data for the in-line scan shown in Figure S1, Si distributions into interlamellar space, where there is usually an amorphous phase of PEG, are present in all composites, except for TP15. A smoother surface, without obvious lamellar morphology, was observed for TP15, as shown in Figure S1b. The melting of the PEG component due to the SEM electron gun was not ruled out in the latter case. An uneven pore structure disrupted the lamellar structure of the PEG component.

### 3.4. FT-IR and UV-Raman Analysis

**Figure 5:**
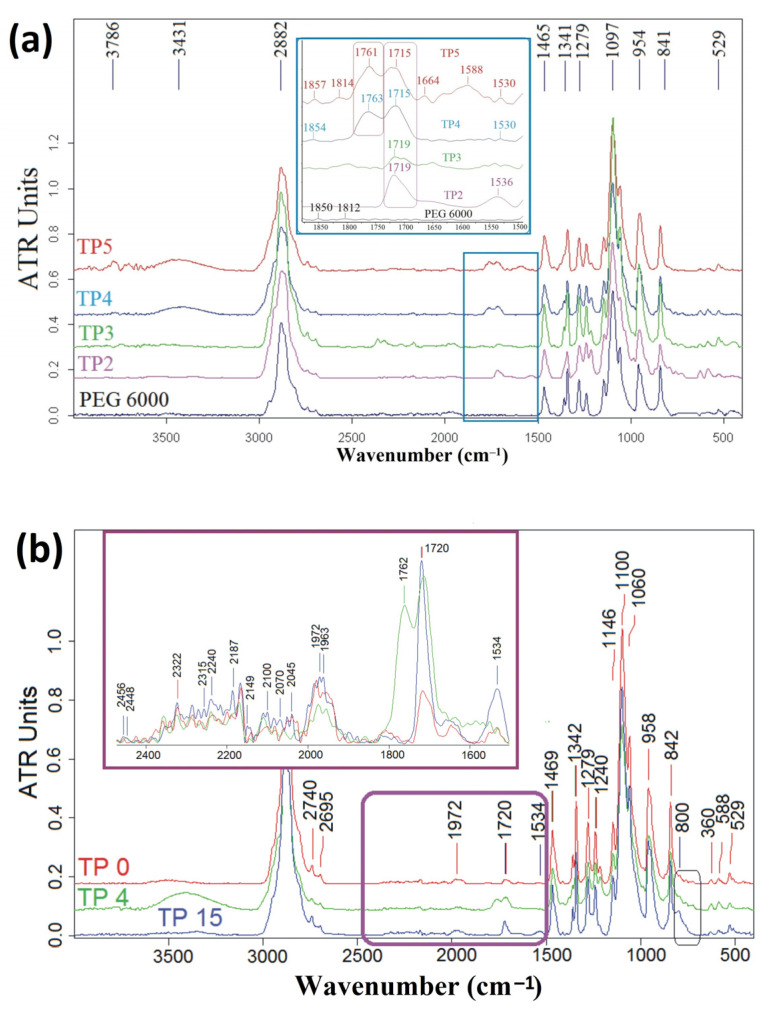
ATR FT-IR spectra of (**a**) TP(2/3/4/5) and PEG_6000_ and (**b**) PT(0/4/15) composites obtained with the same PEG_6000_/NCOTEOS ratio of 2/1 and increasing MWCNTs-OH content.

Since the share of the inorganic silica network in the final composites is small (below 25%), the FTIR spectra of all samples shown in Figure 5 were dominated by the signals coming from PEG. Small differences between the spectra were observed only in the range of 1500 to 1800 cm^−1^ (see the inset of Figure 5 and Figure S2 and Table S1).

Thus, the peaks at ~1719 cm^−1^ (C=O vibration) and ~1530 cm^−1^ (N–H vibration), which are attributed to the newly created urethane bonds (-OCONH-) from isocyanate group (O=C=N–) of the silane coupling agent (NCOTEOS) and the hydroxyl group of PEG, HO-(CH_2_–CH_2_–O)_n_– [55] and/or MWCNTs-OH [56] (radical, R, in the reaction bellow), were clearly visible, especially for the samples in which the PEG weight was lower. These signals appeared for all TP(2/3/4/5) samples. The functionalization of the PEG chains and/or the MWCNTs with triethoxyxilane groups [55] represented a first step in the further sol–gel synthesis of the shape-stabilized organic–inorganic hybrids. The ATR FTIR was a very suitable technique in monitoring this step [55]. No isocyanate vibrations at about 2270 cm^−1^ [55] are depictable in Figure 5.

R–OH + OCN–(CH_2_)_3_–Si(OCH_2_CH_3_)_3_ → R–OCONH-(CH_2_)–Si(OCH_2_CH_3_)_3_ (2)

Two signals at ~1760 cm^−1^ and 1664 cm^−1^ (both carbonyl vibrations), observed for samples with richer PEG content (Figure 5a), might have indicated partial oxidation of the organic components. It was surprising that neither samples TP2 and TP3 nor pure PEG_6000_ exhibited this signal. Their presence also indicated formation beside the urethane bonds of amide (1664 cm^−1^) and anhydride bonds, (RC(O))_2_O (1760 cm^−1^ and 1814 cm^−1^, respectively). In addition, the band at ~1590 cm^−1^ could be assigned to stretching vibrations of the ester groups (O–C=O). The signal at 3431 cm^−1^, which denoted the presence of unreacted hydroxyl groups, was readily discernible for PEG–richer samples, while the peak at 2882 cm^−1^ was present for all samples and was assigned to C−H stretching from the PEG chains. The strongest ether vibrations, at about 1100 cm^−1^ [57], originated from PEG_6000_ (Figure 5). Three main bands were expected for CNTs at ~3430, 1700, and 1180 cm^−1^ (OH, C=O in carboxyl, and C-O bonds, respectively) [58]. The Si-O-Si symmetric stretching vibrations [34] were noticeable as a shoulder of the TP15 spectrum at about 800 cm^−1^, as shown in Figure 5b.

**Figure 6:**
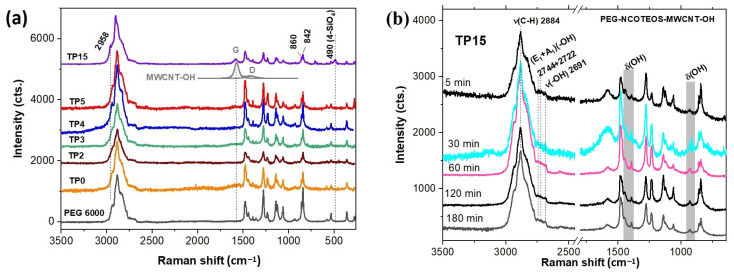
UV-Raman spectra of the TP(0/2/3/4/5/15) composites, PEG_6000_, and fitted D and G bands of the MWCNT-OH (**a**), and PT15 sample after various time spans of reaction before sol–gel process (**b**).

UV-Raman spectroscopy is a very sensitive technique for the investigation of the sample surface, especially for functionalized sp^2^ carbon materials. The thermal and electrical behavior of the CNTs is highly influenced by their interaction with the embedding matrix. The UV-Raman spectra of all composites prepared in this work, as shown in Figure 6, were dominated by the spectral features of the PEG_6000_ [46,59]. No bands for MWCNTs [60] are shown in Figure 6b except for the G band at ~1580 cm^−1^ (in-plane vibrations of the sp^2^ carbon atoms) and the 708 cm^−1^ band with unknown origin [60] in the richer MWCNT containing sample TP15. The defect band, D, of the MWCNTs, located at about 1417 cm^−1^, was masked by the stronger bands of PEG_6000_. Unlike the G band of the sp^2^ hybridized carbon materials, the position and intensity of the D band (the breathing mode of the sp^2^-carbon atoms in the rings) are dependent on the wavelength of the exciting laser [60,61]. In addition, the defect band, D_1_ (4-SiO_4_ rings), was noticeable in the Raman spectrum of the MWCNT-rich sample TP15 [62]. Unfortunately, the band at about 530 cm^−1^ had multiple assignments, e.g., C–C–O bending vibrations of the PEG chains and –C–Si–O– bonds [55]. Due to the distinct spectral features of the TP15 composite, Raman spectra (Figure 6b) of the samples containing NCOTEOS and MWCNTs-OH in a PEG_6000_ environment (see Scheme 1 part A) were collected at various time spans (5, 30, 60, 120, and 180 min). In addition, this sample had the same PEG_6000_/NCOTEOS ratio as the TP4 sample (Table 1) Intriguingly, during the first 60 min, the G band of the MWCNT-OH underwent broadening. Thus, this band accommodated three bands (1562, 1609, and 1660 cm^−1^ bands in Figure S2 with full width at the half maximum, FWHM, of 79, 80, and 125 cm^−1^, respectively). It seems that the silane coupling agent reacted first with hydroxyl groups of the MWCNTs-OH. This explains the poor PEG leakage behavior in TP15. Conversely, the NCOTEOS for the TP5 with 0.02% MWCNTs-OH load was insufficient to covalently bind a large quantity of PEG_6000_ (PEG_6000_/NCOTEOS = 3/1) for a shape-stabilized composite. The position of the stretching and bending modes of the hydroxyl groups at 2691 cm^−1^ and 1692 cm^−1^ (PEG_6000_) [46,63] were not affected during the NCOTEOS coupling (Figure 6b). The covalent and non-covalent interaction of CNTs with the matrix could be identified by some Raman spectral features, such as the position of the D and G bands and their intensity ratio, I_D_/I_G_. This ratio increased from 0.14 in the case of pristine MWCNTs-OH to 0.99 for PT15–60 min. (Figure S3). In the latter sample, the intensity of the 840 and 860 cm^−1^ bands did not correspond to the solid PEG_6000_.

The shape and intensity recovery for the crystallinity band at 844 cm^−1^ of PEG_6000_, attributable to the stretching vibrations of the C-O bonds and the rocking vibrations of the CH_2_ [59], took place in the sample collected after 120 min of reaction. Heating and lowering of the molecular weight of PEGs were two causes of the diminishing intensity of the 844 and 863 cm^−1^ bands. The spectra for the PT15–120 min and 180 min samples were alike, meaning that the process was complete in two hours.

### 3.5. Monitoring the Thermal Stability of the ssCPCMs

**Figure 7:**
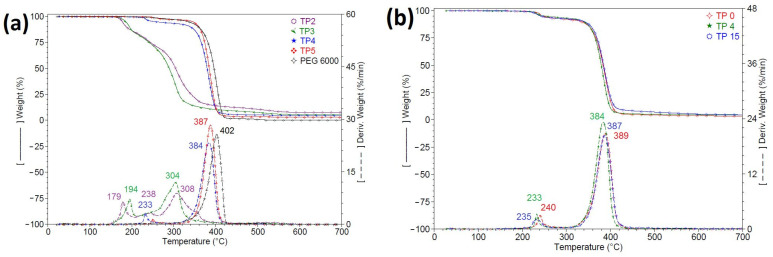
TGA and DTG curves of (**a**) TP(2/3/4/5) composites and PEG_6000_ and (**b**) TP(0/4/15) composites with the same PEG_6000_/NCOTEOS ratio.

TGA and DTG analysis of the TP(0/2/3/4/5/15) composites, as shown in Figure 7, enabled the obtaining of the thermal decomposition range and the temperatures of the maximum decomposition. A slight improvement in the weight was noticeable (Figure 7) for the TP(2/3/4/5/15) composites’ different content of PEG integrated in the silica matrix, in contrast with pure PEG_6000_. Different thermal degradation intervals are visible on the TGA curves. According to the DTG curves shown in Figure 7, the composites with the lowest PEG_6000_ content, e.g., TP2 and TP3, started the degradation process at 170.4 and 179 °C, respectively. For the other materials, the starting degradation temperature increased in succession: TP15 (217.8 °C) < PEG_6000_ (223.5 °C) < TP4 (226.3 °C) < TP0 (230.9 °C) < TP5 (217.8 °C). The first DTG peak was below 200 °C (Figure 7a) and was due to removal of physically adsorbed water and alcohols [39,64] generated by completion of the hydrolysis and by condensation reactions to obtaining the silica.

Therefore, the mass loss in the first interval for samples TP2 and TP3, in which PEG_6000_ was present in only a percentage of 75% and 85%, respectively, was 16–18% (Table 4). A relatively small amount of mass was lost (0.4–0.7%) in samples where the polyethylene glycol component predominated (TP4–91.5% and TP5–94%).

In the range of 215 to 260 °C, hydrolysis continued with the formation and evaporation of ethanol and methanol and condensation occurred with removal of water. However, the dissociation of the PEG-inorganic network urethane bonds, which was accompanied by the release of carbon dioxide and isocyanate, was the primary cause of the mass loss during this thermal interval [64,65].

Between 260 and 450 °C, the largest mass loss (58–97%) took place. Pielichowski et al. [66] reported a single step for thermal degradation of polyethylene oxide, at about 400 °C, as was noticed for the DTG curves of the rich-PEG_6000_ samples, e.g., TP(0/4/5/15). Chemisorbed water from the Si-OH groups can be lost in the same stage [39]. For samples TP2 and TP3, the contribution of the organic functions of the silica co-precursors (having shorter molecular chains than PEG) to the mass loss in this range was significant. In addition, the lower crystallinity degree of the TP2 and TP3 samples underscored that the amorphous phase amount of semicrystalline PEG was bigger. Hence, they were prone to lower thermal stability, when compared to samples TP4 and TP5.

Above 450 °C, partial oxidation of MWCNTs and a possible degradation of residual Si–OH occurred. The differences that were observed between the theoretical and experimental values of the inorganic residues (i.e., silica, SiO_2_) can be explained by the inhomogeneity of the samples taken for the thermogravimetric analysis. They could be extracted from the PEG-rich or PEG-poor regions, which caused a small difference between the experimentally determined weight of the inorganic component and the theoretically predicted one. The sample with the largest discrepancy between the value of the inorganic residue as found experimentally and the value as calculated theoretically was the TP2 sample.

### 3.6. Evaluation of Thermal Diffusivity, Specific Heat, Thermal Conductivity, and Effusivity for PEG_6000_–Silica-MWCNTs PCM Composites

The thermophysical properties of the PEG_6000_-containing materials were dependent on composition, microstructure (crystalline/amorphous phases, chain flexibility/rigidity, etc. [63]), homogeneity and density of the material, and, last but not least, the processing route.

**Figure 8:**
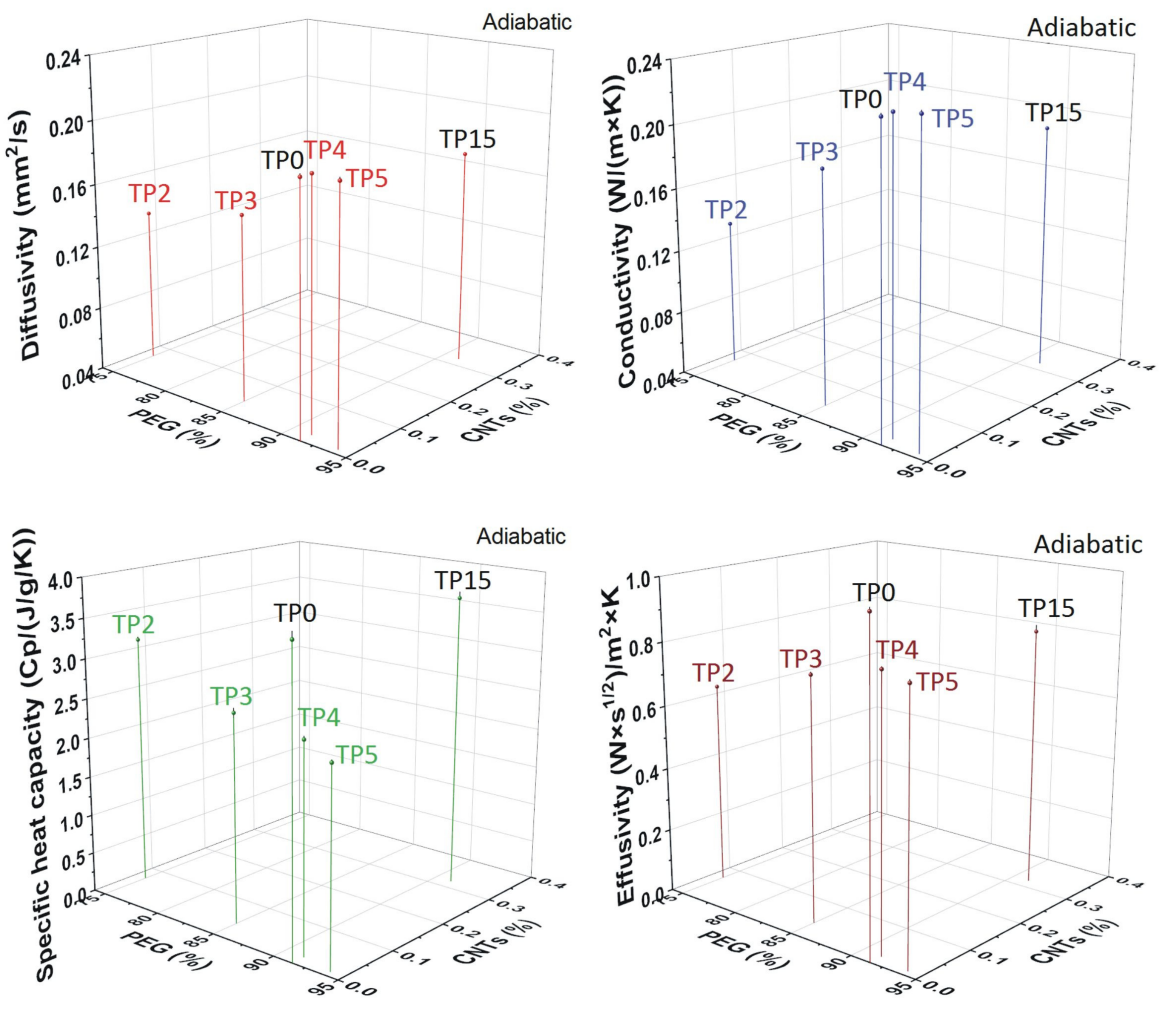
Diffusivity, thermal conductivity, Cp, and effusivity measured by flash method and analyzed by an Adiabatic mathematical model for the TP(0/2/3/4/5/15) samples versus PEG 6000 and MWCNTs-OH content.

It was already known that silica is a poor thermal conductor. Therefore, with its incorporation into the in-situ-generated silica matrix, PEG_6000′_s thermophysical characteristics were found to diminish, as shown in Table 5 and Figure 8.

Moreover, temperature, CNT loading, and sizes (area/volume ratio) were few major parameters influencing thermal conductivity and, hence, heat discharging in functionalized CNTs/PEG materials [67]. However, the TP(2/3/15) encountered lower thermal conductivity values than TP0—i.e., the MWCNTs-free sample. In addition, the compacted MWCNTs-OH had a rather small value in comparison with the value for longitudinal direction [30]. The misoriented MWCNTs, partially covalently linked, did not seem to cause an increase in thermal conductivity. Among the shape-stabilized composites presented here, the TP4 sample had the biggest thermal conductivity, which was a rather low value. Enhancement of the thermal conductivity of the shape stabilized PEG_6000_–SiO_2_-MWCNTs-OH composites remained as a problem to solve in future work.

Thermal effusivity of the rich PEG-containing samples TP(0/4/5/15), i.e., the heat-absorption (charging) rate from the environment [46], had enhanced values compared with PEG_6000_ and TP(2/3). A linear relationship between the amount of PEG in these PCM composite and effusivity existed. Thermal effusivity influences thermal stresses and strains during transient heat conduction and plays a key role in thermal fatigue and thermal shock.

The dispersion and concentration of CNTs also influence the C_p_ values of the PEG/CNTs composites [67]. However, the Cp value of TP4, shown in Table 5, was not intermediate in relation to the ones of TP0 and TP15, meaning that the agglomeration and poor wettability of the MWCNTs by the PCM component (in this case PEG_6000_) did not comply with the rule of the MWCNTs’ content.

### 3.7. Evaluation of the Mechanical Properties of Materials with Stabilized-Shape and Thermal-Energy Storage Properties (Dynamic Mechanical Analysis (DMA))

In order to carry out the DMA analysis, all the samples were pressed in the form of disks in an FTIR mold under high pressure.

**Figure 9:**
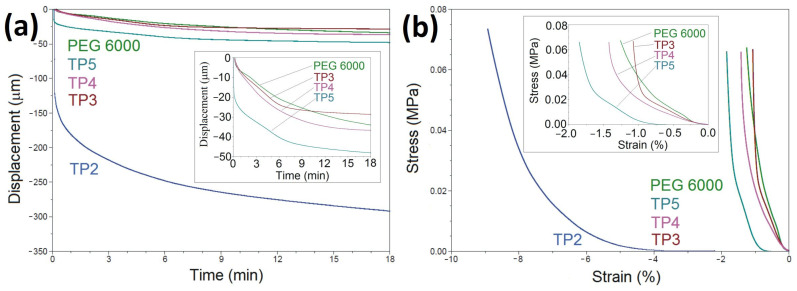
DMA representations of the compression tests recorded for the heat-storage materials, depending on the PEG content of the material: (**a**) displacement over time and (**b**) compression strain (for better visibility, the insets present plots of samples, except for TP2, which was excluded because it behaved very differently from the rest of the set).

Sample TP4 was a homogeneous composite material, in which the presence of the inorganic silica network helped to preserve mechanical properties comparable to those of pure PEG. In addition, PEG was present in a sufficiently large amount to allow the formation of the crystalline component in a weight close to that of pure PEG. The best mechanical properties were presented by the TP3 sample, due to the slightly higher weight of the inorganic network in the final material, compared to that of the TP4 sample (Figure 9). However, the presence of silica in a higher percentage affected the other properties of interest for thermal-energy storage (thermal conductivity, effusivity, and transition enthalpies). In contrast, sample TP5 exhibited weaker mechanical properties than TP4 because of an insufficient inorganic network to reinforce the material.

In turn, sample TP2 had too much inorganic component, which led to an inhomogeneous distribution of PEG and silica in the composite material and a lack of adhesion between these components, phenomena that made the sample crumbly after pressing. This sample, therefore, presented the weakest mechanical properties.

### 3.8. Thermal Reliability and Reusability

For sample TP4, with a PEG_6000_/NCOTEOS = 2/1 molar ratio, it was shown above that the material retains its shape after exposure to high temperature (~80 °C for 2 h). At the same time, the values of the melting and solidification enthalpies maintain high values (146.1 J/g for the melting stage and 145.1 J/g for the solidification stage, respectively). These thermal properties make the TP4 material a very good candidate for thermal-energy storage applications. As a result, this sample was selected for the cycling test, which provides information about the material’s long-term behavior after repeated uses.

**Figure 10:**
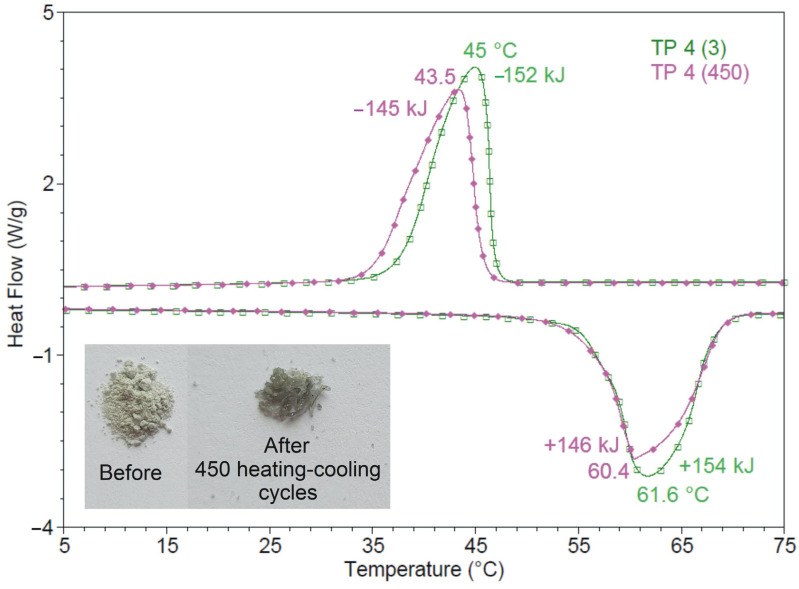
DSC runs for the third and 450th heating–cooling cycles of the TP4 sample.

The DSC method was used to assess the long-term stability of TP4 ssPCM, and 450 repeated melting–solidification cycles of were performed. The third and 450th heating-cooling cycles (Figure 10) were utilized to estimate the changes in the sample’s thermal characteristics between the test’s initial moment and its ending. We aimed to eliminate the thermal history of the sample; therefore, the first two heating–cooling cycles were not considered. In addition, Figure S5 (Supplementary Materials) in the supporting information provides a visual representation of an overlay representing each of the 450 heating–cooling cycles.

The melting temperatures (T_mo_ and T_mp_) and the crystallization temperatures (T_fo_ and T_fp_) recorded for sample TP4, as shown in Table 6, were very close after 450 melting–crystallization cycles. As expected, after 450 consecutive heating–cooling cycles, the phase-transition enthalpies for the melting stage (H_m_) and the cooling stage (H_f_), respectively, showed slight decreases (8 J/g and 7 J/g, respectively).

Coefficients ƞ_m_ and ƞ_f_, which represent the magnitude of change in the phase-transition enthalpies as a result of the cycling test [68], accounted for 5.2% and 4.8% decrease, respectively in enthalpies values (Table 6). There were no noticeable visual changes in the sample after cycling.

Therefore, from the point of view of the thermal conductivity and the capacity of the material to transfer heat, and from the point of view of the stabilization of the shape and the homogeneity of the final composite, the TP4 sample presented the best characteristics. In addition, it exhibited a high phase-transition capacity (87%) compared to that of pure PEG, preserving high values of transition enthalpies (~150 J/g).

## 4. Conclusions

The new ssCPCM developed in the current work (sample TP4) was obtained via a chemical procedure (an in situ sol–gel process) and included PEG_6000_ as the phase-changing material, in-situ-generated silica as the inorganic stabilizing structure, and MWCNTs as the thermoconductive fillers. Both a part of the PEG chains and a part of the MWCNTs-OH were covalently connected by newly formed urethane bonds to the in-situ-generated silica matrix in order to prevent the PEG from flowing out of the structure in which it was integrated. For this innovative PEG-silica-MWCNTs ssCPCM, various physico–chemical characterization techniques were evaluated. Thus, the novel material, which contains 91.5% PEG_6000_ after drying, exhibited a solid–liquid phase transition, with melting occurring in the temperature range of 55 °C to 70 °C, with the maximum at ~61 °C, and solidification occurring in the temperature range of 30 °C to 45 °C, with the maximum at ~42 °C. Its determined relative enthalpy efficiency was 87%.

To verify its thermal reliability and reusability, TP4 composite PCM was subjected to 450 complete successive heating–cooling cycles, with no leakage of the melted PEG from the composite structure being observed. During this test, the melting (146.1 J/g) and solidification (145.1 J/g) enthalpy preserved high values, compared to those of the pure PEG (~187.63 J/g).

These properties demonstrated that the composite PCM we created has the required thermal behavior for use in solar energy storage units to shield some exposed construction elements from overheating, or to prevent electrical equipment, such as batteries, processors, engines, and other devices, from overheating. In future studies, the optimization of the amount and orientation of the multi- and single-wall CNT thermoconductive filler in order to improve the thermal conductivity of the composites, while maintaining the thermal energy storage performance, will be considered. In the next work, light-to-heat conversion will be tested, together with thermal-energy storage for the optimized compositions.

## 5. Patents

A patent application entitled “Process for the preparation of a product with thermal energy storage properties based on a PEG_6000_-silica-carbon nanotubes composite material with stabilized shape” (a 2022 00629/13.10.2022), resulting from the work reported in this manuscript, was submitted to O.S.I.M. Romania (the State Office for Inventions and Trademarks).

## Figures and Tables

**Table 1:** Composition of the PEG-silica shape-stabilized PCM materials.

Sample Code	PEG_6000_/NCOTEOS Molar Ratio	PEG_6000_ % wt.	Silica % wt.	MWCNTs-OH % wt.
TP2	1/2	75	10.58	0.07
TP3	1/1	85	6.11	0.04
TP15	2/1	91.23	3.30	0.32
TP4	2/1	91.51	3.30	0.02
TP0	2/1	91.53	3.30	0
TP5	3/1	94	2.25	0.01

**Table 2:** Composition of the A, B, and C reaction mixtures for the synthesis of PEG-based ssCPCMs.

Sample Code	A (PEG Mixture)					B (Solvent)	C (Sol–Gel Precursors Mixture)				
	PEG_6000_ (g)	DABCO (g)	MWCNTs (g)	NCOTES (g)	SDS (g)	n-octan (mL)	TEOS (g)	GPTMS (g)	MeTMS (g)	EtOH (mL)	NH_4_OH (mL)
TP2	22	0.2	0.02	2	4	80	5	0.567	0.327	20	1.5
TP3	43.2	0.43	0.02								
TP15	86	0.86	0.306								
TP4	86	0.86	0.02								
TP0	86	0.86	0								
TP5	130	1.3	0.02								

**Table 3:** Evaluation of the thermal energy storage capacity for the obtained materials.

Sample Code	w_PEG_	T_mo_	T_m_	ΔH_m_	^1^ χ_c_ (DSC)	^2^ XRD	T_fo_	T_f_	ΔH_f_	λ_m_	λ_f_	^3^ ΔT
	(%)	(°C)	(°C)	(J/g)	%	%	(°C)	(°C)	(J/g)	(%)	(%)	(°C)
TP2	75	48.9	56.0	92.64	67.17	69.44	43.5	41.2	−91.37	65.83	66.77	14.8
TP3	85	55.9	60.6	134.65	80.41	82.84	44.0	42.3	−131.32	84.43	84.68	18.3
TP15	91.24	50.8	54.4	141.76	78.87	75.92	41.9	41.2	−137.16	82.81	82.40	13.3
TP4	91.51	56.7	60.9	150.10	83.27	82.35	44.5	43.2	−146.54	87.42	87.77	17.7
TP0	91.53	56.2	60.4	153.04	84.87	87.86	41.5	40.9	−149.31	89.12	89.41	19.5
TP5	94	58.5	61.5	169.81	91.69	92.24	44.4	43.2	−165.98	96.28	96.78	18.3
PEG_6000_	100	59.4	61.4	187.63	94.77		43.4	42.5	−182.45	-	-	18.9

^1^ χ_c_ = percent of crystallized PEG in PCM, calculated from DSC analyses; χ_c_ = ΔH_m_ × (w_PEG6000_ × ΔH0)^−1^ × 100, where ΔH_0_ of 197 kJ/Kg corresponds to latent heat of fully crystallized PEG [48]. ^2^ XRD = percent of crystallized PEG in PCM, calculated from XRD analyses. ^3^ ΔT = subcooling range. The standard uncertainty for melting temperature and enthalpy were estimated to be 0.01 °C and 0.02·ΔH_m_ from the range of 25 to 75 °C.

**Table 4:** Weight loss (Wt loss) and corresponding temperature ranges.

Sample Code	0–215 °C		215–260 °C		260–450 °C		450–700 °C		^1^ Rezidue (exp) 700 °C	^2^ Rezidue (theo) 700 °C
	Wt loss	Tmax	Wt loss	Tmax	Wt loss	Tmax	Wt loss	Tmax		
	(%)	(°C)	(%)	(°C)	(%)	(°C)	(%)	(°C)	(%)	(%)
TP2	16.97	178.7	12.53	238.4	58.28	307.7	4.79	527.7	7.43	10.65
TP3	17.84	194.3	14.04	242.1	59.24	303.9	3.42	518.9	5.46	6.15
TP15	1.17	-	5.39	234.9	85.25	387.4	3.70	-	4.49	3.62
TP4	0.93	-	4.52	233.2	89.07	383.7	1.15	-	4.33	3.32
TP0	0.84	-	5.31	240.3	89.00	388.6	1.54	-	3.31	3.30
TP5	0.42	-	1.51	250.8	94.54	387.4	0.66	-	2.87	2.26
PEG_6000_	0.68	-	0.96	-	96.67	402.4	1.69	-	0	0

^1^ Experimental residue (measured); ^2^ Theoretical residues (calculated).

**Table 5:** The values of the thermophysical properties evaluated by the adiabatic model for PEG-silica-MWCNTs composites.

Sample Code	PEG_6000_ %	MWCNT%	*ρ* ± U (g/cm^3^)	*α* ± U (mm^2^/s)	*C_p_* ± U (J/g∙K)	*λ* ± U (W/m∙K)	*e* ± U (W∙s^1/2^/m^2^∙K)
MWCNT	0	100	0.495 ± 0.001	0.708 ± 0.091	1.650 ± 0.169	0.343 ±0.004	0.522 ± 0.026
TP2	75	0.07	0.973 ± 0.002	0.136 ± 0.001	3.153 ± 0.039	0.132 ± 0.001	0.637 ± 0.006
TP3	85	0.04	1.198 ± 0.002	0.156 ± 0.001	2.611 ± 0.060	0.186 ± 0.001	0.764 ± 0.009
TP15	91.23	0.32	1.131 ± 0.004	0.176 ± 0.001	3.702 ± 0.080	0.195 ± 0.001	0.819 ± 0.021
TP4	91.51	0.02	1.187 ± 0.008	0.193 ± 0.001	2.568 ± 0.043	0.229 ± 0.001	0.836 ± 0.008
TP0	91.53	0	1.182 ± 0.008	0.194 ± 0.002	3.707 ± 0.093	0.228 ± 0.002	1.003 ± 0.012
TP5	94	0.01	1.199 ± 0.002	0.193 ± 0.002	2.425 ± 0.034	0.232 ± 0.002	0.822 ± 0.010
PEG_6000_	100	0	1.200 ± 0.002	0.225 ± 0.009	1.780 ± 0.034	0.270 ± 0.011	0.759 ± 0.022
Pyroceram 9606	-	-	2.545	2.4158 ± 0.004	0.800	4.1	2.889

U = measurement’s uncertainty.

**Table 6:** Thermal parameters for sample TP4 after 3 and 450 heating–cooling cycles.

Sample TP4	T_mo_	T_mp_	ΔH_m_	^1^ ƞ_m_	T_fo_	T_fp_	ΔH_f_	^2^ ƞ_f_
	(°C)	(°C)	(J/g)	(%)	(°C)	(°C)	(J/g)	(%)
Cycle 3	57.3	61.6	154.1		46.7	45.0	−152.4	
Cycle 450	56.6	60.4	146.1	5.2	45.7	43.3	−145.1	4.8

^1^ η_m_ = melting efficiency = (ΔH_m_(cycle 3) − ΔH_m_(cycle 450))/ΔH_m_(cycle 3) × 100. ^2^ η_f_ = crystallization efficiency = (ΔH_f_(cycle 3) − ΔH_f_(cycle 450))/ΔH_f_(cycle 3) × 100 (calculated as in ref. [68]).

## Data Availability

Not applicable.

## Supplementary Materials

The following supporting information can be downloaded at: https://www.mdpi.com/article/10.3390/polym15143022/s1. Figure S1. SEM micrographs (left), EDX Scanning Line (middle) and EDX Line Sum Spectrum (right) for: (a) samples with increasing amount of PEG_6000_ (Series TP2, TP3, TP4 and TP5) and (b) samples with increasing amount of MWCNTs-OH (series TP0, TP4 and TP15. Figure S2. FT-IR spectra of sample TP2 during the synthesis, at time t0 (time of NCOTEOS feeding) and at time t1 (after 2 h from NCOTEOS feeding. Table S1. Peak position and assignments of the FT-IR spectra within the 1500–1900 cm^−1^ range. Figure S3. FittedUV-Raman spectra within 750–1850 cm^−1^ range of the PEG 6000, MWCNTs-OH, and TP15 during the synthesis, after 30 min from NCOTEOS feeding. Table S2. Thermophysical properties of the obtained PEG-silica-MWCNTs composites, evaluated by the Cowan model. Figure S4. Modification of thermophysical properties depending on the content of PEG and MWCNTs-OH in the material, analyzed by the Cowan mathematical model. Figure S5 DSC diagrams corresponding to the 450 successive heating-cooling cycles (left) and modification of the thermal parameters during the 450 melting cycles (right). Table S3. The evolution of the values of the thermal parameters recorded for sample TP4 during the 450 successive heating-cooling cycles.

## Nomenclature

ssCPCMs = shape-stabilized composite phase change materials;
MWCNTs-OH = multi-wall carbon nanotubes functionalized with hydroxyl groups;
NCOTEOS = 3-(Triethoxysilyl)propyl isocyanate;
PEG_6000_-Si = PEG_6000_ coupled with NCOTEOS (newly created building block for the synthesis of the 3D network);
MWCNTs-Si = MWCNTs-OH coupled with NCOTEOS;
TEOS = tetraethylorthosilicate;
GPTMS = (3-Glycidyloxypropyl)trimethoxysilane;
MeTMS = methyltrimethoxysilane;
w_PEG6000_ = percentage of PEG_6000_ in the final material;
λ_m_ = phase-transition capacity for the heating stage;
ΔH_m_ = experimental melting enthalpy (latent heat) of ssPCM;
ΔH_mPEG6000_ = melting enthalpy of pure PEG_6000_;
λ_f_ = phase-transition capacity for the cooling stage;
ΔH_f_ = experimental crystallization enthalpy (latent heat) of ssPCM;
ΔH_sPEG6000_ = solidification enthalpy of pure PEG_6000_;
T_m_ = temperature at which the melting rate is maximum;
T_f_ = temperature at which the rate of crystallization is maximum;
α = thermal diffusivity;
Cp = specific heat capacity;
ρ = density of ssPCM composite;
λ = thermal conductivity;
e = thermal effusivity;
T_mo_ = onset melting temperature for cycling tests;
T_mp_ = temperature at which the melting rate is maximum for cycling tests;
η_m_ = melting efficiency = (ΔH_m_(cycle 450) − ΔH_m_(cycle 3))/ΔH_m_(cycle 3) × 100;
T_fo_ = onset crystallization temperature for cycling tests;
T_fp_ = temperature at which the crystallization rate is maximum for cycling tests;
η_f_ = crystallization efficiency = (ΔH_f_(cycle 450) − ΔH_f_(cycle 3))/ΔH_f_(cycle 3) × 100.

## References

[1] Diaconu B.M., Cruceru M., Anghelescu L. (2022). Phase Change Materials—Applications and Systems Designs: A Literature Review. Designs.

[2] Salgado-Pizarro R., Padilla J.A., Xuriguera E., Barreneche C., Fernández A.I. (2021). Novel Shape-Stabilized Phase Change Material with Cascade Character: Synthesis, Performance and Shaping Evaluation. Energies.

[3] Li Z., Shahsavar A., Al-Rashed A.A., Talebizadehsardari P. (2020). Effect of Porous Medium and Nanoparticles Presences in a Counter-Current Triple-Tube Composite Porous/Nano-PCM System. Appl. Therm. Eng..

[4] Yang H., Bai Y., Ge C., He L., Liang W., Zhang X. (2022). Polyethylene Glycol-Based Phase Change Materials with High Photothermal Conversion Efficiency and Shape Stability in an Aqueous Environment for Solar Water Heater. Compos. Part A Appl. Sci. Manuf..

[5] Zhang X., Xu M., Liu L., Liu L., Wang M., Ji H., Song K.-I. (2020). The Concept, Technical System and Heat Transfer Analysis on Phase-Change Heat Storage Backfill for Exploitation of Geothermal Energy. Energies.

[6] Sun K., Kou Y., Zheng H., Liu X., Tan Z., Shi Q. (2018). Using Silicagel Industrial Wastes to Synthesize Polyethylene Glycol/Silica-Hydroxyl Form-Stable Phase Change Materials for Thermal Energy Storage Applications. Sol. Energy Mater. Sol. Cells.

[7] Ferfera R.S., Madani B. (2020). Thermal Characterization of a Heat Exchanger Equipped with a Combined Material of Phase Change Material and Metallic Foams. Int. J. Heat Mass Transf..

[8] Weiss L., Jha R. (2023). Small-Scale Phase Change Materials in Low-Temperature Applications: A Review. Energies.

[9] Gürel B. (2020). A Numerical Investigation of the Melting Heat Transfer Characteristics of Phase Change Materials in Different Plate Heat Exchanger (Latent Heat Thermal Energy Storage) Systems. Int. J. Heat Mass Transf..

[10] Koytsoumpa E.I., Bergins C., Kakaras E. (2018). The CO2 Economy: Review of CO2 Capture and Reuse Technologies. J. Supercrit. Fluids.

[11] Ali H.M. (2019). Applications of Combined/Hybrid Use of Heat Pipe and Phase Change Materials in Energy Storage and Cooling Systems: A Recent Review. J. Energy Storage.

[12] Feng Z., Li Y., He F., Li Y., Zhou Y., Yang Z., He R., Zhang K., Yang W. (2020). Experimental and Numerical Simulation of Phase Change Process for Paraffin in Three-Dimensional Graphene Aerogel. Appl. Therm. Eng..

[13] Ji R., Wei S., Xia Y., Huang C., Huang Y., Zhang H., Xu F., Sun L., Lin X. (2020). Enhanced Thermal Performance of Form-Stable Composite Phase-Change Materials Supported by Novel Porous Carbon Spheres for Thermal Energy Storage. J. Energy Storage.

[14] Wang J., Wu Z., Xie H., Wang T., Wang Y., Huang Y., Dong L. (2022). Effect of Different Soft Segment Contents on the Energy Storage Capacity and Photo–Thermal Performance of Polyurethane-Based/Graphene Oxide Composite Solid–Solid Phase Change Materials. Polymers.

[15] Zhou T., Ma X., Yuan J., Zheng N., Sun Z. (2020). Moving Solid-Liquid Interface-Based Measurement Method for Thermal Conductivity Determination of Phase Change Materials in Liquid Phase. Exp. Therm. Fluid Sci..

[16] Deng Y., He M., Li J., Yang Z. (2018). Polyethylene Glycol-Carbon Nanotubes/Expanded Vermiculite Form-Stable Composite Phase Change Materials: Simultaneously Enhanced Latent Heat and Heat Transfer. Polymers.

[17] Sundararajan S., Samui A.B., Kulkarni P.S. (2017). Shape-Stabilized Poly(Ethylene Glycol) (PEG)-Cellulose Acetate Blend Preparation with Superior PEG Loading via Microwave-Assisted Blending. Sol. Energy.

[18] Kumar A., Kulkarni P.S., Samui A.B. (2014). Polyethylene Glycol Grafted Cotton as Phase Change Polymer. Cellulose.

[19] Vélez J.F., Aparicio M., Mosa J. (2016). Covalent Silica-PEO-LiTFSI Hybrid Solid Electrolytes via Sol-Gel for Li-Ion Battery Applications. Electrochim. Acta.

[20] Lin W., Wang F., Wang H., Li H., Fan Y., Chan D., Chen S., Tang Y., Zhang Y. (2022). Thermal-Stable Separators: Design Principles and Strategies Towards Safe Lithium-Ion Battery Operations. ChemSusChem.

[21] Baskaran K., Ali M., Gingrich K., Porter D.L., Chong S., Riley B.J., Peak C.W., Naleway S.E., Zharov I., Carlson K. (2022). Sol-Gel Derived Silica: A Review of Polymer-Tailored Properties for Energy and Environmental Applications. Microp. Mesopor. Mater..

[22] Ghalambaz M., Shahabadi M., Mehryan S.A.M., Sheremet M., Younis O., Talebizadehsardari P., Yaici W. (2021). Latent Heat Thermal Storage of Nano-Enhanced Phase Change Material Filled by Copper Foam with Linear Porosity Variation in Vertical Direction. Energies.

[23] Zahir M.H., Rahman M.M., Irshad K., Shaikh M.N., Helal A., Aziz M.A., Ali A., Khan F. (2022). Energy Conversion Efficiency Enhancement of Polyethylene Glycol and a SiO_2_ Composite Doped with Ni, Co, Zn, and Sc Oxides. ACS Omega.

[24] Serrano A., Borreguero A.M., Iglesias I., Acosta A., Rodríguez J.F., Carmona M. (2021). Diffusion of Shape Stabilized PEG-SiO_2_ as a Driver for Producing Thermoregulating Facing Bricks. Materials.

[25] Atinafu D.G., Wang C., Dong W., Chen X., Du M., Gao H., Wang G. (2020). In-Situ Derived Graphene from Solid Sodium Acetate for Enhanced Photothermal Conversion, Thermal Conductivity, and Energy Storage Capacity of Phase Change Materials. Sol. Energy Mater. Sol. Cells.

[26] Liu L., Su D., Tang Y., Fang G. (2016). Thermal Conductivity Enhancement of Phase Change Materials for Thermal Energy Storage: A Review. Renew. Sust. Energy Rev..

[27] Cabaleiro D., Hamze S., Fal J., Marcos M.A., Estellé P., Żyła G. (2020). Thermal and Physical Characterization of PEG Phase Change Materials Enhanced by Carbon-Based Nanoparticles. Nanomaterials.

[28] Marcos M.A., Podolsky N.E., Cabaleiro D., Lugo L., Zakharov A.O., Postnov V.N., Charykov N.A., Ageev S.V., Semenov K.N. (2019). MWCNT in PEG-400 Nanofluids for Thermal Applications: A Chemical, Physical and Thermal Approach. J. Mol. Liq..

[29] Tang B., Wang Y., Qiu M., Zhang S. (2014). A full-band sunlight-driven carbon nanotube/PEG/SiO_2_ composites for solar energy storage. Sol. Energy Mater. Sol. Cells.

[30] Chen X., Cheng P., Tang Z., Xu X., Gao H., Wang G. (2021). Carbon-Based Composite Phase Change Materials for Thermal Energy Storage, Transfer, and Conversion. Adv. Sci..

[31] Donchak V., Stetsyshyn Y., Bratychak M., Broza G., Harhay K., Stepina N., Kostenko M., Voronov S. (2021). Nanoarchitectonics at surfaces using multifunctional initiators of surface-initiated radical polymerization for fabrication of the nanocomposites. Appl. Surf. Sci. Adv..

[32] Yang Y., Pang Y., Liu Y., Guo H. (2018). Preparation and Thermal Properties of Polyethylene Glycol/Expanded Graphite as Novel Form-Stable Phase Change Material for Indoor Energy Saving. Mater. Lett..

[33] Gao J., Tao W., Chen D., Guo X., Chen Y., Jiang Y. (2018). High Performance Shape-Stabilized Phase Change Material with Nanoflower-Like Wrinkled Mesoporous Silica Encapsulating Polyethylene Glycol: Preparation and Thermal Properties. Nanomaterials.

[34] Min X., Fang M., Huang Z., Liu Y., Huang Y., Wen R., Qian T., Wu X. (2015). Enhanced Thermal Properties of Novel Shape-Stabilized PEG Composite Phase Change Materials with Radial Mesoporous Silica Sphere for Thermal Energy Storage. Sci. Rep..

[35] Nguyen G.T. (2023). Polyethylene Glycol/Fumed Silica Composites as Shape-Stabilized Phase Change Materials with Effective Thermal Energy Storage. RSC Adv..

[36] Mokhtari S., Madhkhan M. (2022). The Performance Effect of PEG-Silica Fume as Shape-Stabilized Phase Change Materials on Mechanical and Thermal Properties of Lightweight Concrete Panels. Case Stud. Constr. Mater..

[37] Mohaisen K.O., Zahir H., Maslehuddin M. (2023). Shape-stabilized phase change material for thermal energy storage: Sr^+2^ doped BaCO_3_ matrix incorporating polyethylene glycol. J. Energy Storage.

[38] He L., Li J., Zhou C., Zhu H., Cao X., Tang B. (2014). Phase Change Characteristics of Shape-Stabilized PEG/SiO_2_ Composites Using Calcium Chloride-Assisted and Temperature-Assisted Sol Gel Methods. Sol. Energy.

[39] Serrano A., Martín Del Campo J., Peco N., Rodriguez J.F., Carmona M. (2019). Influence of Gelation Step for Preparing PEG–SiO_2_ Shape-Stabilized Phase Change Materials by Sol–Gel Method. J. Sol-Gel Sci. Technol..

[40] Yang H., Feng L., Wang C., Zhao W., Li X. (2012). Confinement Effect of SiO_2_ Framework on Phase Change of PEG in Shape-Stabilized PEG/SiO_2_ Composites. Eur. Polym. J..

[41] Wang W., Yang X., Fang Y., Ding J., Yan J. (2009). Enhanced Thermal Conductivity and Thermal Performance of Form-Stable Composite Phase Change Materials by Using β-Aluminum Nitride. Appl. Energy.

[42] Wang W., Yang X., Fang Y., Ding J. (2009). Preparation and Performance of Form-Stable Polyethylene Glycol/Silicon Dioxide Composites as Solid–Liquid Phase Change Materials. Appl. Energy.

[43] Weng Z., Wu K., Luo F., Xiao F., Zhang Q., Wang S., Lu M. (2018). Fabrication of High Thermal Conductive Shape-Stabilized Polyethylene Glycol/Silica Phase Change Composite by Two-Step Sol Gel Method. Compos. Part A Appl. Sci. Manuf..

[44] Mishra D.K., Bhowmik S., Pandey K.M. (2020). Polyethylene Glycol Based Form Stable Composite Phase Change Material: A Review. J. Phys. Conf. Ser..

[45] Tao Y.B., Lin C.H., He Y.L. (2015). Effect of Surface Active Agent on Thermal Properties of Carbonate Salt/Carbon Nanomaterial Composite Phase Change Material. Appl. Energy.

[46] Anghel E.M., Pavel P.M., Constantinescu M., Petrescu S., Atkinson I., Buixaderas E. (2017). Thermal transfer performance of a spherical encapsulated PEG 6000-based composite for thermal energy storage. Appl. Energy.

[47] (2017). Standard Test Method for Thermal Diffusivity by the Flash Method.

[48] Zhang L., Wang Y.-Z., Yang K.-K., Wang X.-L., Chen S.-C., Li J. (2005). Effect of PEG on the crystallization of PPDO/PEG blends. Eur. Polym. J..

[49] Li J., He L., Liu T., Cao X., Zhu X. (2013). Preparation and characterization of PEG/SiO_2_ composites as shape-stabilized phase change materials for thermal energy storage. Sol. Energy Mater. Sol. Cells.

[50] Yan D., Ming W., Liu S., Yin G., Zhang Y., Tang B., Zhang S. (2021). Polyethylene glycol (PEG)/silicon dioxide grafted aminopropyl group and carboxylic multi-walled carbon nanotubes (SAM) composite as phase change material for light-to-heat energy conversion and storage. J. Energy Storage.

[51] Yu C., Xie Q., Bao Y., Shan G., Pan P. (2017). Crystalline and Spherulitic Morphology of Polymers Crystallized in Confined Systems. Crystals.

[52] Li Y., Ma Q., Huang C., Liu G. (2013). Crystallization of Poly (Ethylene Glycol) in Poly (Methyl Methacrylate) Networks. Mater. Sci..

[53] Wang K., Wen R. (2023). Scaphium scaphigerum/graphene hybrid aerogel for composite phase change material with high phase change enthalpy and high thermal conductivity for energy storage. J. Energy Storage.

[54] Kong Y., Hay J.N. (2001). The enthalpy of fusion and degree of crystallinity of polymers as measured by DSC. Eur. Polym. J..

[55] Ficher D., Pospiech D., Scheler U., Navarro R., Messori M., Fabbri P. (2008). Monitoring of the sol-gel synthesis of Organic-Inorganic Hybrids by FTIR Transmission, FTIR/ATR, NIR and Raman Spectroscopy. Macromol. Symp..

[56] Luo X., Yu Z., Cai Y., Wu Q., Zeng J. (2019). Facile Fabrication of Environmentally-Friendly Hydroxyl-Functionalized Multiwalled Carbon Nanotubes/Soy Oil-Based Polyurethane Nanocomposite Bioplastics with Enhanced Mechanical, Thermal, and Electrical Conductivity Properties. Polymers.

[57] Raj C.R., Suresh S., Vasudevan S., Chandrasekar M., Singh V.K., Bhavsar R.R. (2020). Thermal performance of nano-enriched form-stable PCM implanted in a pin finned wall-less heat sink for thermal management application. Energy Conver. Manag..

[58] Salam M.A., Burk R. (2017). Synthesis and characterization of multi-walled carbon nanotubes modified with octadecylamine and polyethylene glycol. Arab. Chem. J..

[59] Samuel A.Z., Umapathy S. (2014). Energy funneling and macromolecular conformational dynamics: A 2D Raman correlation study of PEG melting. Polym. J..

[60] Ravindran T.R., Jackson B.R., Badding J.V. (2001). UV Raman Spectroscopy of Single-Walled Carbon Nanotubes. Chem. Mater..

[61] Zhang H.-B., Lin G.-D., Zhou Z.-H., Dong X., Chen T. (2002). Raman spectra of MWCNTs and MWCNT-based H_2_-adsorbing system. Carbon.

[62] Aguiar H., Serra J., González P., León B. (2009). Structural study of sol–gel silicate glasses by IR and Raman spectroscopies. J. Non-Cryst. Solid.

[63] Pielichowska K., Glowinkowski S., Lekki J., Binias D., Pielichowski K., Jenczyk J. (2008). PEO/fatty acid blends for thermal energy storage materials. Structural/morphological features and hydrogen interactions. Eur. Polym. J..

[64] Hernández-Escolano M., Ramis X., Jiménez-Morales A., Juan-Díaz M., Suay J. (2012). Study of the Thermal Degradation of Bioactive Sol–Gel Coatings for the Optimization of Its Curing Process. J. Therm. Anal. Calorim..

[65] Jiao L., Xiao H., Wang Q., Sun J. (2013). Thermal Degradation Characteristics of Rigid Polyurethane Foam and the Volatile Products Analysis with TG-FTIR-MS. Polym. Degrad. Stab..

[66] Pielichowski K., Flejtuch K. (2005). Non-oxidative thermal degradation of poly(ethylene oxide): Kinetic and thermoanalytical study. J. Anal. Appl. Pyrolysis.

[67] Manasrah A.D., Laoui T., Zaidi S.J., Atieh M.A. (2017). Effect of PEG functionalized carbon nanotubes on the enhancement of thermal and physical properties of nanofluids. Exp. Therm. Fluid Sci..

[68] Lu X., Fang C., Sheng X., Zhang L., Qu J. (2019). One-Step and Solvent-Free Synthesis of Polyethylene Glycol-Based Polyurethane As Solid–Solid Phase Change Materials for Solar Thermal Energy Storage. Ind. Eng. Chem. Res..

