# Supplementary material for: Novel PEG6000–Silica-MWCNTs Shape-Stabilized Composite Phase-Change Materials (ssCPCMs) for Thermal-Energy Storage
Source: Polymers (Basel). 2023 Jul 12;15(14):3022. doi: 10.3390/polym15143022 (PMC10386010; doi:10.3390/polym15143022)
Supplement: Supplementary file 1 [file polymers-15-03022-s001.zip › polymers-2431534-supplementary.pdf]

polymers

Supplementary Information for:

Novel PEG_{6000}–Silica-MWCNTs Shape-Stabilized Composite Phase-Change Materials (ssCPCMs) for Thermal-Energy Storage

Cristina Lavinia Nistor [1], Ioana Catalina Gifu [1], Elena Maria Anghel [2,*], Raluca Ianchis [1], Cristiana-Diana Cirstea [3], Cristian Andi Nicolae [1], Augusta Raluca Gabor [1], Irina Atkinson [2] and Cristian Petcu [1,*]

1 Polymers Department, National Institute for Research and Development in Chemistry and Petrochemistry-ICECHIM, 202 Spl. Independentei, 060021 Bucharest, Romania; cristina.nistor@icechim-pd.ro (C.L.N.); catalina.gifu@icechim-pd.ro (I.C.G.); raluca.ianchis@icechim-pd.ro (R.I.); cristian.nicolae@icechim.ro (C.A.N.); raluca.gabor@icechim.ro (A.R.G.)
2 Institute of Physical Chemistry "Ilie Murgulescu" of the Romanian Academy, 202 Splaiul Independentei, 060021 Bucharest, Romania; irinaatkinson@yahoo.com
3 National Institute for Research and Development in Electrical Engineering ICPE-CA, INCDIE ICPE-CA, 313 Splaiul Unirii Street, 030138 Bucharest-3, Romania; diana.cirstea@icpe-ca.ro
* Correspondence: eanghel@hotmail.com (E.M.A.); cristian.petcu@icechim-pd.ro (C.P.); Tel.: 021-312-34-93 (107) (C.P.)

The results of SEM-EDX investigation are presented in Figure S1.

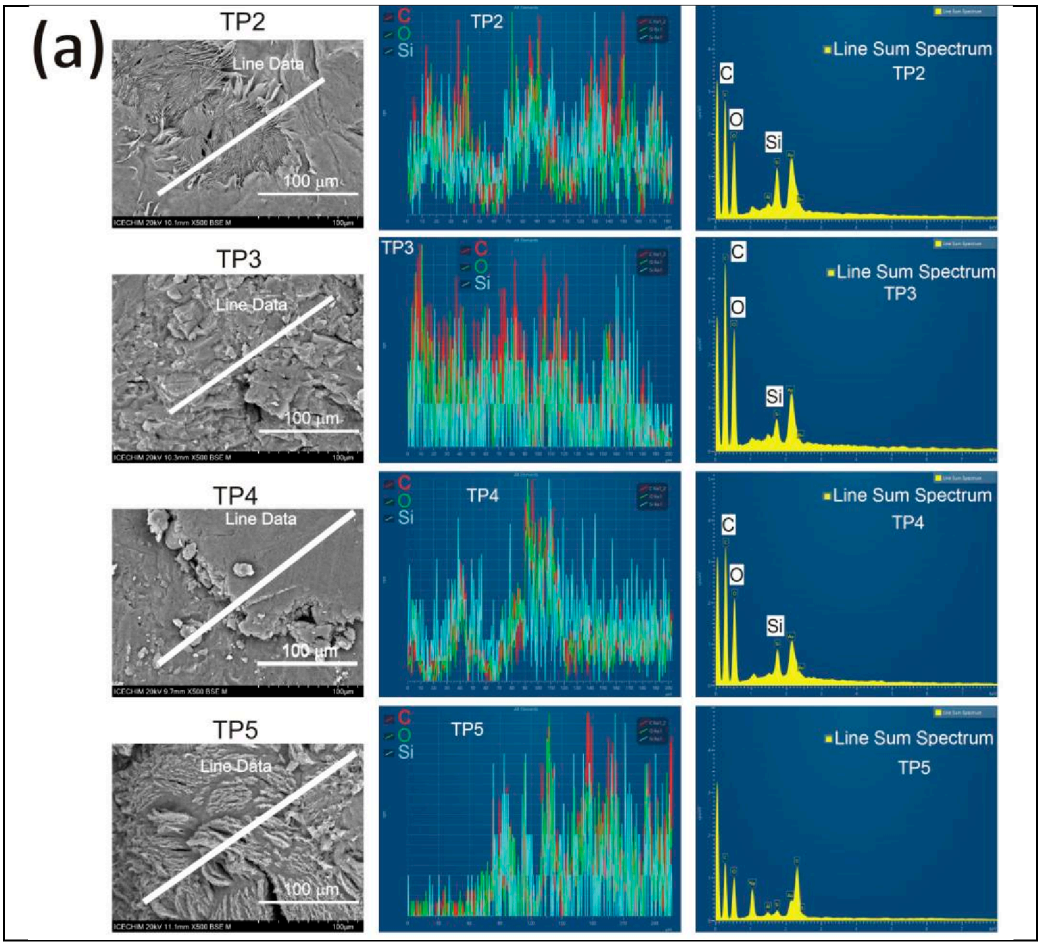

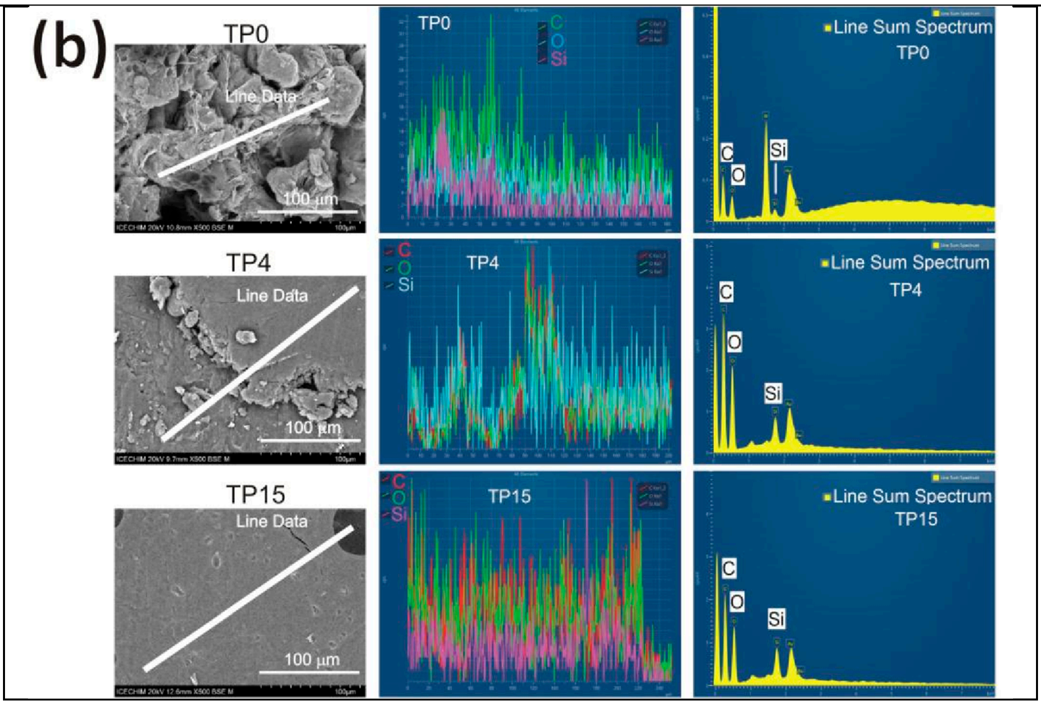

Figure S1. SEM micrographs (left), EDX Scanning Line (middle) and EDX Line Sum Spectrum (right) for: **a)** samples with increasing amount of PEG6000 (Series TP2, TP3, TP4 and TP5) and **b)** samples with increasing amount of MWCNTs-OH (series TP0, TP4 and TP15

Monitoring the formation of the urethane bonds (the coupling reaction between PEG_{6000} and NCOTEOS) was achieved by FT-IR spectroscopy. In Figure S2, FT-IR spectra of the TP2 sample during the synthesis, at time t_0 (time of NCOTEOS feeding) and at time t_1 (after 2h from NCOTEOS feeding) are depicted.

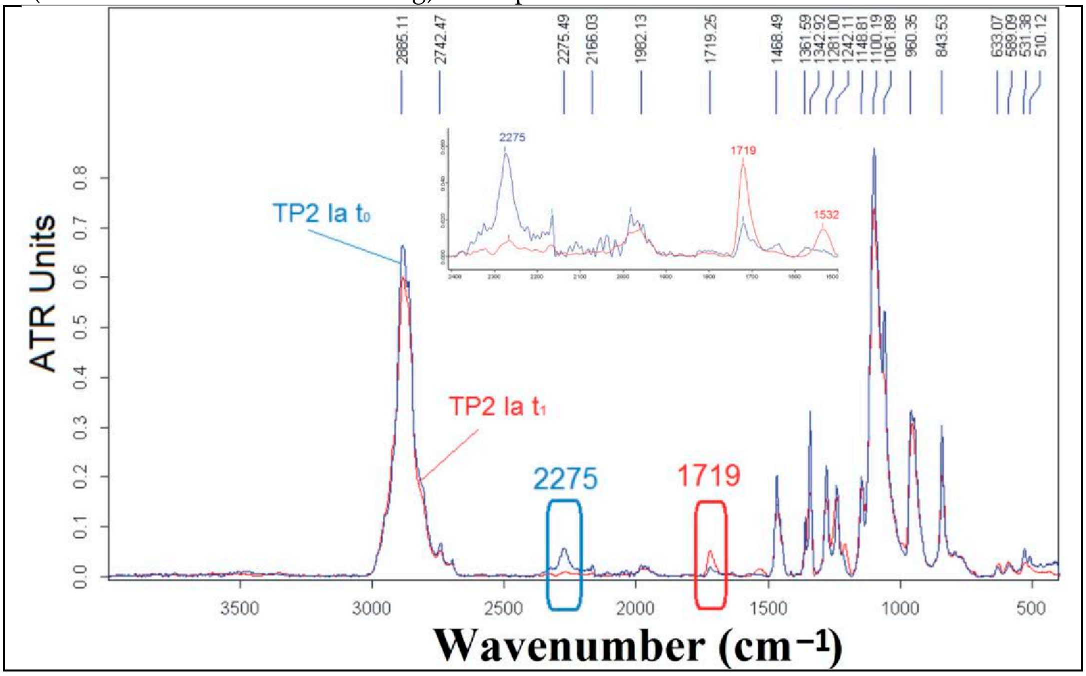

Figure S2. FT-IR spectra of sample TP2 during the synthesis, at time t0 (time of NCOTEOS feeding) and at time t1 (after 2h from NCOTEOS feeding

Table S1. Peak position and assignments of the FT-IR spectra within the 1500-1900 cm^{-1} range.

Peak position (cm^{-1})							Assignement	Refrence
TP15	TP5	TP4	TP3	TP2	TP0	PEG_{6000}		
	1857	1854				1850		
	1814	1814	1814			1812	Anhydride bonds, (RC(O))2O	57
	1761	1763					carbonyl vibrations	55
1720	1715	1715	1719	1719			C=O vibration from polyurethan link and C=C bonds from MWCNT	55,56,58
	1664						carbonyl vibrations	56
	1588						Ester group (asymmetric stretching of COO^-)	57,59
1520	1530	1530	1530	1536			N-H vibration	56

56. Ficher, D.; Pospiech, D.; Scheler, U.; Navarro, R.; Messori, M.; Fabbri, P. Monitoring of the sol-gel synthesis of Organic-Inorganic Hybrids by FTIR Transmission, FTIR/ATR, NIR and Raman Spectroscopy. *Macromol. Symp.* **2008**, *265*, 134–143. https://doi.org/10.1002/masy200850514
57. Luo, X.; Yu, Z.; Cai, Y.; Wu, Q.; Zeng, J. Facile Fabrication of Environmentally-Friendly Hydroxyl-Functionalized Multiwalled Carbon Nanotubes/Soy Oil-Based Polyurethane Nanocomposite Bioplastics with Enhanced Mechanical, Thermal, and Electrical Conductivity Properties. *Polymers* **2019**, *11*, 763. https://doi.org/10.3390/polym11050763.
58. Raj, C.R.; Suresh, S.; Vasudevan, S.; Chandrasekar, M.; Singh, V.K.; Bhavsar, R.R. Thermal performance of nano-enriched form-stable PCM implanted in a pin finned wall-less heat sink for thermal management application. *Energy Conver. Manag.* **2020**, *226*, 113466. https://doi.org/10.1016/j.enconman.2020.113466.
59. Salam, M.A.; Burk, R. Synthesis and characterization of multi-walled carbon nanotubes modified with octadecylamine and polyethylene glycol. *Arab. Chem. J.* **2017**, *10*, S921–S927. https://doi.org/10.1016/j.arabjc.2012.12.028.

Also, the coupling reaction between both PEG6000 and MWCNTS-OH with NCOTEOS was studied by Raman spectroscopy.

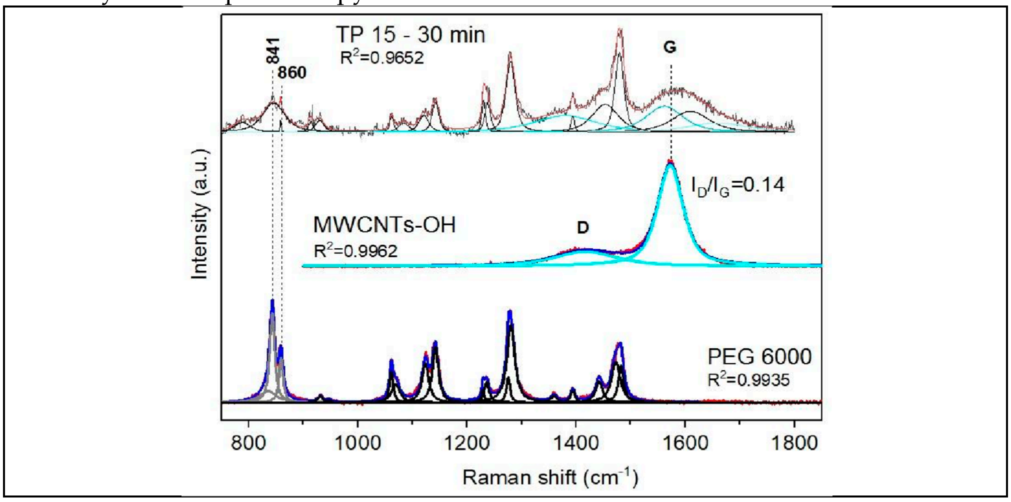

Figure S3. FittedUV-Raman spectra within 750-1850 cm^{-1} range of the PEG 6000, MWCNTs-OH, and TP15 during the synthesis, after 30 minutes from NCOTEOS feeding.

Table S2. Thermophysical properties of the obtained PEG-silica-MWCNTs composites, evaluated by the Cowan model.

Sample code	PEG_{6000} %	MWCNT %	[1.]ρ ± U (g/cm^3)	[2.]α± U (mm^2/s)	[3.]C_p± U (J/g·K)	[4.]λ± U (W/m·K)	[5.]e±[6]U (W·s$^{1/2}$/m^2·K)
**MWCNT**	0	100	0.495 ± 0.001	0.495 ±0.001	1.650 ± 0.169	0.239 ± 0.001	0.436 ± 0.023
**TP2**	75	0.07	0.973 ± 0.002	0.085 ± 0.001	1.704 ± 0.045	0.089 ± 0.001	0.384 ± 0.006
**TP3**	85	0.04	1.198 ± 0.002	0.113 ± 002	1.627 ± 0.069	0.137 ± 0.003	0.517 ± 0.016
**TP15**	91.23	0.32	1.131 ± 0.004	0.138 ± 0.001	3.702 ± 0.080	0.153 ± 0.001	0.794 ± 0.016
**TP4**	91.51	0.02	1.187 ± 0.008	0.139 ± 0.002	1.594 ± 0.025	0.165 ± 0.002	0.559 ± 0.007
**TP0**	91.53	0	1.182 ± 0.008	0.143 ± 0.003	3.751 ± 0.152	0.169 ± 0.003	0.865 ± 0.024
**TP5**	94	0.01	1.199 ± 0.002	0.140 ± 0.001	1.55 ± 0.014	0.175 ± 0.001	0.571 ± 0.004
**PEG_{6000}**	100	0	1.200 ± 0.002	0.180 ± 0.003	1.399 ± 0.020	0.209 ± 0.004	0.592 ± 0.009
**Pyroceram 9606**	-	-	2.545	1.985 ± 0.010	0.799	4.04	2.866

[1]ρ = sample density- the average of measurements on three replicas per sample; [2.] = thermal diffusivity; [3.]C_p = specific heat capacity; [4.]λ = thermal conductivity; [5.]e = thermal effusivity; [6.]U = measurement's uncertainty.

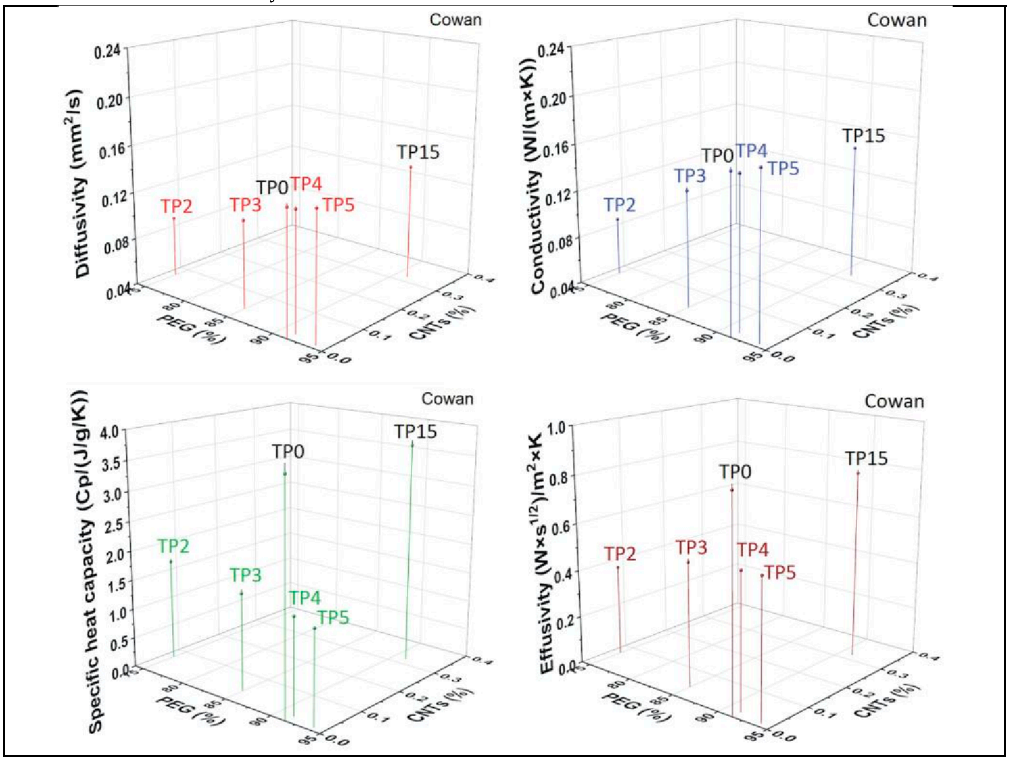

Figure S4. Modification of thermophysical properties depending on the content of PEG and MWCNTs-OH in the material, analyzed by the Cowan mathematical model

The results recorded for sample TP4 during 450 heating cooling cycles are presented in Figure S5 and Table S3.

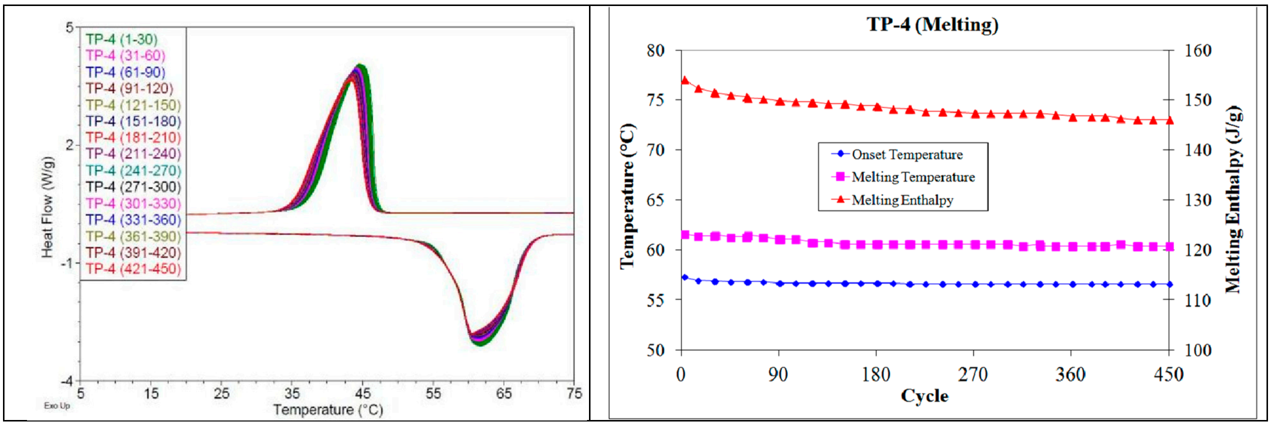

Figure S5 DSC diagrams corresponding to the 450 successive heating-cooling cycles (left) and modification of the thermal parameters during the 450 melting cycles (right)

Table S3. The evolution of the values of the thermal parameters recorded for sample TP4 during the 450 successive heating-cooling cycles

Cycle	[a]Tmo (°C)	[b]Tmp (°C)	[c]ΔHm (J/g)	[d]Tfo (°C)	[e]Tfp (°C)	[f]ΔHf (J/g)
3	57.3	61.6	+154.1	46.7	45.0	-152.4
15	57.0	61.4	+152.4	46.3	44.5	-151.0
30	56.9	61.4	+151.5	46.2	44.1	-150.1
31	56.9	61.5	+151.4	46.1	44.2	-150.0
45	56.8	61.3	+151.0	46.1	44.0	-149.8
60	56.8	61.3	+150.7	46.0	44.0	-149.3
61	56.8	61.3	+150.4	46.0	44.0	-149.1
75	56.8	61.3	+150.2	46.0	43.9	-148.9
90	56.7	61.1	+149.8	45.9	43.9	-148.4
91	56.7	61.1	+149.8	45.9	43.9	-148.4
105	56.7	61.1	+149.7	45.9	43.9	-148.0
120	56.7	60.8	+149.6	45.9	43.6	-148.2
121	56.7	60.8	+149.6	45.9	43.6	-148.2
135	56.7	60.8	+149.3	45.8	43.7	-148.1
150	56.7	60.6	+149.2	45.8	43.6	-148.1
151	56.7	60.6	+149.2	45.8	43.6	-148.1
165	56.7	60.6	+148.8	45.8	43.6	-147.7
180	56.7	60.6	+148.8	45.8	43.4	-147.4
181	56.7	60.6	+148.7	45.8	43.6	-147.4
195	56.7	60.6	+148.3	45.8	43.6	-147.4
210	56.6	60.6	+148.2	45.7	43.6	-147.1
211	56.6	60.6	+148.2	45.7	43.6	-147.0
225	56.6	60.6	+147.7	45.8	43.3	-146.8
240	56.6	60.6	+147.7	45.8	43.5	-146.4
241	56.6	60.6	+147.7	45.7	43.5	-146.4
255	56.6	60.6	+147.5	45.7	43.5	-146.4
270	56.6	60.6	+147.4	45.7	43.3	-146.2
271	56.6	60.6	+147.4	45.7	43.5	-146.0
285	56.6	60.6	+147.4	45.7	43.5	-146.0
300	56.6	60.6	+147.4	45.7	43.3	-146.0

301	56.6	60.6	+147.4	45.7	43.3	-146.0
315	56.6	60.6	+147.4	45.7	43.3	-146.0
330	56.6	60.6	+147.4	45.7	43.3	-146.0
331	56.6	60.6	+147.4	45.7	43.3	-146.0
345	56.6	60.4	+147.1	45.7	43.3	-145.7
360	56.6	60.4	+146.7	45.7	43.3	-145.6
361	56.6	60.4	+146.7	45.7	43.3	-145.6
378	56.6	60.4	+146.7	45.7	43.3	-145.6
390	56.6	60.4	+146.7	45.7	43.3	-145.5
391	56.6	60.4	+146.7	45.7	43.3	-145.5
405	56.6	60.4	+146.4	45.7	43.3	-145.5
420	56.6	60.4	+146.1	45.7	43.3	-145.1
421	56.6	60.4	+146.1	45.7	43.3	-145.1
435	56.6	60.4	+146.1	45.7	43.3	-145.1
450	56.6	60.4	+146.1	45.7	43.3	-145.1

Note: [a]Onset melting temperature. [b]The temperature at which the melting rate is maximum. [c]ΔH_m = Melting enthalpy. [d]Onset crystallization temperature. [e]The temperature at which the crystallization rate is maximum. [f]ΔH_f = Crystallization enthalpy.

Disclaimer/Publisher's Note: The statements, opinions and data contained in all publications are solely those of the individual author(s) and contributor(s) and not of MDPI and/or the editor(s). MDPI and/or the editor(s) disclaim responsibility for any injury to people or property resulting from any ideas, methods, instructions or products referred to in the content.
